# Close kin influence COVID-19 precautionary behaviors and vaccine acceptance of older individuals

**DOI:** 10.21203/rs.3.rs-1699988/v1

**Published:** 2022-06-02

**Authors:** Bruno Arpino, Valeria Bordone, Giorgio Di Gessa

**Affiliations:** University of Florence; University of Vienna; University College London

## Abstract

The family plays a central role in shaping health behaviors of its members through social control and support mechanisms. We investigate whether and to what extent close kin (i.e., partner and children) have mattered for older people in taking on precautionary behaviors (e.g., physical distancing) and vaccination during the COVID-19 pandemic in Europe. Drawing on data from the Survey of Health, Ageing and Retirement in Europe (SHARE), we combine its Corona Surveys (June-August 2020 and June-August 2021) with pre-COVID information (October 2019-March2020). We find that having close kin (especially a partner) is associated with a higher probability of both adopting precautionary behaviors and accepting a COVID-19 vaccine. Results are robust to controlling for other potential drivers of precautionary behaviors and vaccine acceptance, as well as to accounting for co-residence with kin. Our findings suggest that policy makers and practitioners may differently address kinless individuals when promoting public policy measures.

In the early phases of the COVID-19 pandemic, individual precautionary behaviors were the only weapon to protect people from infection and reduce the spread of the virus in the community. Due to herd immunity remaining a distant target^1^ and given that COVID-19 vaccines neither permanently nor completely protect against infection^2-4^, precautionary health behaviors have remained crucial also after the launch of COVID-19 vaccination campaigns. Thus, governments across the globe have imposed or recommended behaviors such as physical distancing, mask-wearing, and frequent hand-washing. Although these have been presented as general guidelines for everyone, individuals at greater risk of developing a more severe case of COVID-19 when infected by the Coronavirus, such as older individuals, have been particularly encouraged to adopt precautionary health behaviors during all phases of the pandemic in order to limit admissions to Intensive Care Units and deaths from COVID-19-related conditions^5-10^. Monitoring and understanding compliance with COVID-19-preventive behaviors have thus become a prime target for research since the beginning of the pandemic^11-18^.

More recently, the fight against the pandemic has entered a new stage with the approval by health authorities of effective COVID-19 vaccines. Although it is widely recognized that effective and equitable distribution of COVID-19 vaccines is a key policy priority^19-20^, ensuring their acceptance by the population is just as important. Thus, several studies have aimed at understanding the determinants of vaccine acceptance (i.e., actual vaccine intake and intention to be vaccinated)^21-30^.

Despite the acknowledged importance of precautionary health behaviors and vaccines to limit the spread of the virus, compliance with guidelines and vaccine acceptance are anything but universal. Even among older adults, that are at the highest risk of COVID-19 complications, studies have shown that a large share of individuals does not follow the recommended precautionary behaviors^11-12^ and/or is unvaccinated and not willing to be vaccinated^23-26^. Socio-demographic characteristics of individuals such as gender and education, and health conditions have been shown to be associated with both the adoption of precautionary health behaviors^14-18^ and vaccine acceptance^27-30^. For example, highly educated individuals and those in poorer health conditions were more likely to follow the guidelines and get vaccinated, while a gender “paradox” emerged^31^: women are more likely to adopt precautionary behaviors but less likely to accept COVID-19 vaccines. Other studies have considered the role of anti-intellectualisms^32^, science skepticism^33^, information trust^34^ and misinformation^35^. We extend the existing work by analyzing whether having close kin (a partner and/or children) influences the adoption of precautionary health behaviors and vaccine acceptance.

Indeed, social ties are known to influence health behaviors throughout the lifecourse^36-38^. In particular, partner and children tend to represent the most important social ties for older adults in terms of emotional closeness and intensity of support^38-41^. Therefore, numerous studies have investigated the role of having close kin (i.e., a partner and/or children) on health behaviors (see the reviews in [42-44]). The theoretical social-behavioral explanations of the importance of the family for health behaviors focus on the instrumental and emotional support that family members provide to each other complying with social norms of family obligations^45-46^. Family members complement thus the role of the health care system by providing material support, information, and motivation to prevent diseases and help adhering to medical treatments or recommendations^47^.

The power of close kin to improve health is also explained by the social control function of family members, which exert pressures and control to inhibit unhealthy behaviors and to promote positive habits and lifestyles^38,48,49^. Social control affects health behaviors directly (through sanctions for deviant behaviors, regulation, and physical interventions) and indirectly (through internalization of norms of healthful behavior and facilitation of positive health behaviors)^48^. Partnership and parenthood, in particular, enhance a sense of obligation and greater self-regulation that discourage harmful behaviors and boost healthy ones^48,50^. Although under certain circumstances (e.g., family conflicts or multiple roles overload) kin may have a negative effect on health behaviors^47,51^, most studies have found positive effects^38,43^. Moreover, partnership tends to be more consistently beneficial for health and health behaviors as compared to parenthood^48,52,53^. The influence of family members on health behaviors is particularly strong when they live together^48^ and later in life^54^.

In the context of the COVID-19 pandemic, the social control function of partners and children might have been particularly relevant to vehiculate information about the importance of adopting precautionary behaviors (e.g., wearing masks) and of vaccination. Similarly, children might have provided instrumental support to their older parents with (online and in-person) shopping in order to limit their in-person contacts. Thus, we should expect individuals with a partner and children to be more likely to adopt precautionary behaviors and (to be willing) to get vaccinated compared to their counterparts who lack these kin ties. Based on findings from the literature on family and health behaviors mentioned above, the effect should also be stronger for partnership than parenthood status.

We test the role of close kin in precautionary health behaviors and COVID-19 vaccine acceptance using large-scale representative data from the Survey of Health, Ageing and Retirement in Europe (SHARE), a survey on individuals aged 50 or more implemented in several European countries^55^. We combine data from the two SHARE Corona Surveys, administered in June-August 2020 and June-August 2021, with information from the latest pre-COVID wave (regular wave 8; October 2019-March 2020) from 27 countries. Our findings suggest that partnership and parenthood are positively associated with the likelihood of adopting precautionary health behaviors and to accept COVID-19 vaccine. The effect of having a partner is found to be stronger than that of having children. Our results urge policy makers and practitioners in the health sector to pay special attention to kinless individuals when designing interventions and recommendations related to precautionary health behaviors and vaccination. This research provides important insights to be better prepared for the next phases of the COVID-19 pandemic and in case of future pandemics.

## Results

### Having close kin and precautionary behaviors

**Figure 1:**
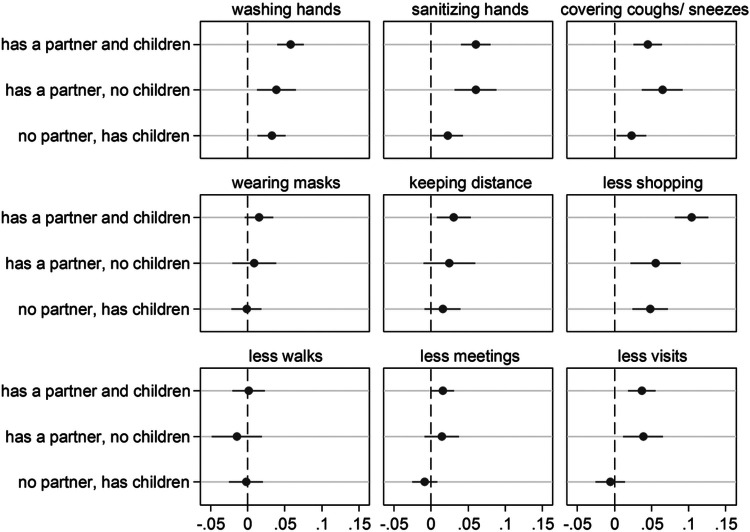
Having close kin (partner and children) and COVID-19 precautionary behaviors Notes: The graph shows the effect of the explanatory variable (having kin) in the form of Average Marginal Effects (AMEs) with 95% confidence intervals from nine separate logistic regression models (one for each of the considered precautionary behaviors). Each AME compares the predicted probability of adopting a precautionary behavior for one of the three groups of older adults who have kin available (e.g., those who have both a partner and children) with the predicted probability of the outcome for the reference group (kinless, i.e. older adults who lack both partner and children). All control variables are included in the models. Full estimates are available in Table S.1 in the Supplementary Materials. Data are from SHARE Corona Survey 1 (June-August 2020).

We first present the results based on the SHARE Corona Survey 1 (SCS1) which collected information on nine precautionary behaviors in June-August 2020. To ease interpretation of results, we present them graphically in Fig. 1 in terms of Average Marginal Effects (AMEs) with 95% confidence intervals obtained from fully-adjusted logistic regression models (see Methods for the socio-demographic and health variables we controlled for). The full table of regression estimates (log-odds) is reported in the Supplementary Materials (Table S.1).

Results in Fig. 1 show that, overall, respondents who have close kin (partner or children) are more likely to adopt the suggested precautionary health behaviors against the spread of the virus compared to kinless older adults. As an example, compared to older people who do not have a partner, partnered older adults (independently of whether they have children or not) are about 6 percentage points more likely to use hand sanitizer or disinfection fluids more frequently than before the outbreak of the pandemic. The positive effect of kin is particularly evident for partnership: for most outcomes, having a *partner and no children* is more often associated with a higher probability of adopting precautionary behaviors than having *children and no partner*. In addition, the AMEs for those who have a *partner and children* are usually very similar and not statistically different to the AMEs for those who have a *partner and no children*. The only precautionary behavior where the combined effect of partnership and parenthood is both significantly and substantially higher than the effect of partnership alone is for reporting less shopping: partnered parents are about 5 percentage points more likely to report having left home for shopping less often or not at all since the outbreak of the pandemic than partnered respondents without children.

### Having close kin and vaccine acceptance

**Figure 2:**
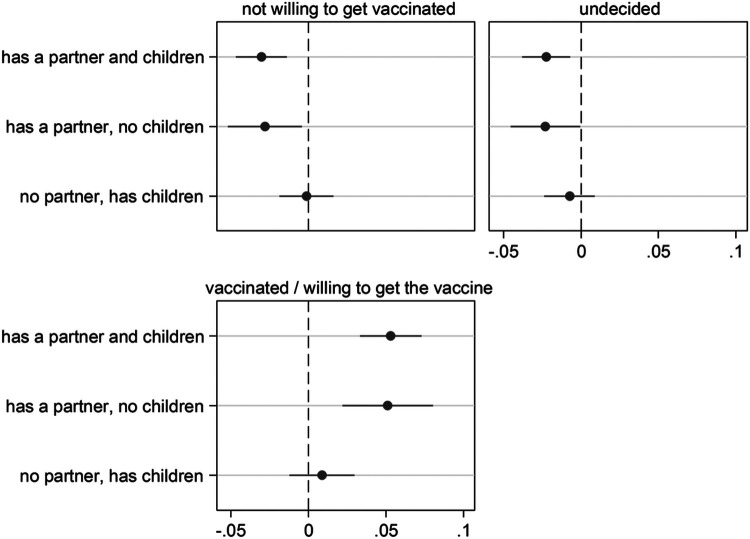
Having close kin (partner and children) and COVID-19 vaccine acceptance Notes: The graph shows results for the effect of the explanatory variable (having kin) in the form of Average Marginal Effects (AMEs) with 95% confidence intervals from a multinomial logistic regression model for the three-level categorical outcome vaccine acceptance. Each AME compares the predicted probability of a certain outcome category (e.g., being vaccinated or willing to get the vaccine) for one of the three groups of older adults who have kin available (e.g., those who have both a partner and children) with the predicted probability for the reference group (kinless, i.e. older adults who lack both partner and children). All control variables are included in the models. Full estimates are available in Table S.2 in the Supplementary Materials. Data are from SHARE Corona Survey 2 (June-August 2021).

Next, we present results about vaccine acceptance based on SHARE Corona Survey 2 (SCS2; June-August 2021), the only SHARE survey where this information is available. Figure 2 presents estimated AMEs (with 95% confidence intervals) obtained from a fully-adjusted multinomial logistic regression (full regression estimates are available in Table S.2 of the Supplementary Materials). Figure 2 shows that the probability of being already vaccinated or planning to do so is about 5 percentage points higher for respondents who have a partner (independently of whether they have children or not). Correspondingly, older adults in a partnership are less likely of both being undecided about vaccination and of not intending to get vaccinated. Parenthood, instead, does not seem to play a role in vaccine acceptance. In fact, the AMEs of having *children and no partner* are very close to zero, and not statistically significant. In addition, the effect of partnership is neither substantially nor statistically modified by its combination with parenthood.

### Heterogeneity analyses

**Figure 3:**
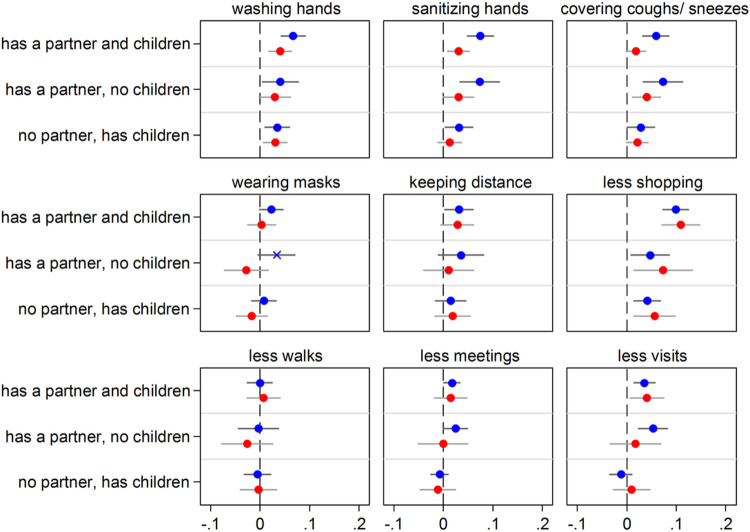
Having close kin (partner and children) and COVID-19 precautionary behaviors by age group (65+ in blue; 50-64 in red) Notes: The graph shows the effect of the explanatory variable (having kin) in the form of Average Marginal Effects (AMEs) with 95% confidence intervals from nine separate logistic regression models (one for each of the considered precautionary behaviors). Each model includes an interaction between the explanatory variable and a dummy variable for age that distinguishes two groups. Thus, separate AMEs by age are obtained (65+ in blue; 50-64 in red). Each AME compares the predicted probability of adopting a precautionary behavior for each one of the three groups of older adults who have kin available (e.g., those who have both a partner and children) with that for the reference group (kinless, i.e. older adults who lack both partner and children). Statistically significant differences (p<0.05) between the AMEs of the two considered age groups are indicated by an “x” in correspondence of the bigger AME. All control variables are included in the models. Full estimates are available in Table S.3 in the Supplementary Materials. Data are from SHARE Corona Survey 1 (June-August 2020).

**Figure 4:**
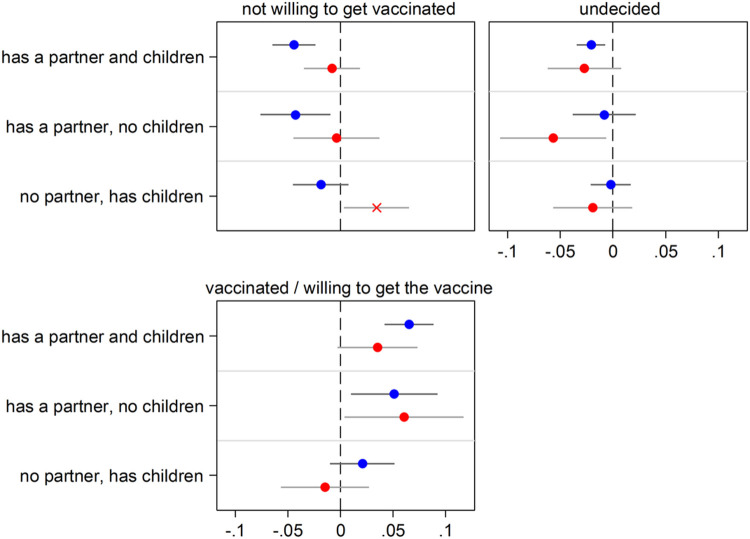
Having close kin (partner and children) and COVID-19 vaccine acceptance by age group (65+ in blue; 50-64 in red) Notes: The graph shows results for the effect of the explanatory variable (having kin) in the form of Average Marginal Effects (AMEs) with 95% confidence intervals from a multinomial logistic regression model for the three-level categorical outcome vaccine acceptance. Each model includes an interaction between the explanatory variable and a dummy variable for age that distinguishes two groups. Thus, separate AMEs by age are obtained (65+ in blue; 50-64 in red). Each AME compares the predicted probability of a certain outcome category (e.g., being vaccinated or willing to get the vaccine) for each one of the three groups of older adults who have kin available (e.g., those who have both a partner and children) with that for the reference group (kinless, i.e. older adults who lack both partner and children). Statistically significant differences (p<0.05) between the AMEs of the two considered age groups are indicated by an “x” in correspondence of the bigger AME. All control variables are included in the models. Full estimates are available in Table S.4 in the Supplementary Materials. Data are from SHARE Corona Survey 2 (June-August 2021).

**Figure 5:**
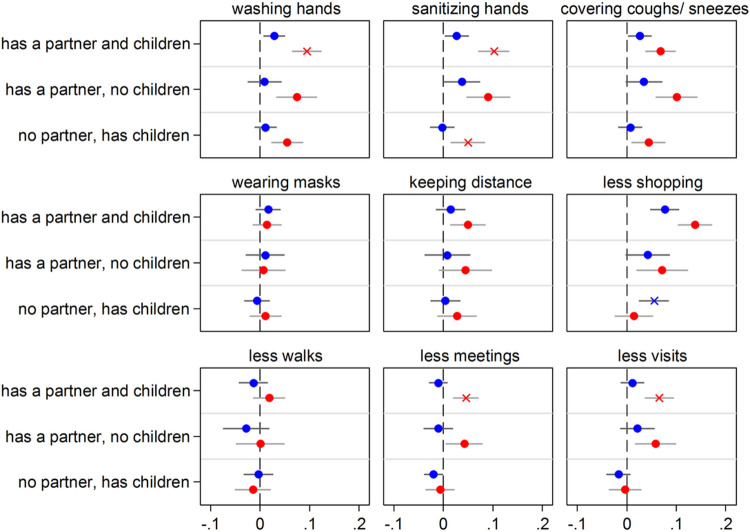
Having close kin (partner and children) and COVID-19 precautionary behaviors by gender (women in blue; men in red) Notes: The graph shows the effect of the explanatory variable (having kin) in the form of Average Marginal Effects (AMEs) with 95% confidence intervals from nine separate logistic regression models (one for each of the considered precautionary behaviors). Each model includes an interaction between the explanatory variable and a dummy variable for gender. Thus, separate AMEs by gender are obtained (women in blue; men in red). Each AME compares the predicted probability of adopting a precautionary behavior for each one of the three groups of older adults who have kin available (e.g., those who have both a partner and children) with that for the reference group (kinless, i.e. older adults who lack both partner and children). Statistically significant differences (p<0.05) between the AMEs of the two genders are indicated by an “x” in correspondence of the bigger AME. All control variables are included in the models. Full estimates are available in Table S.5 in the Supplementary Materials. Data are from SHARE Corona Survey 1 (June-August 2020).

**Figure 6:**
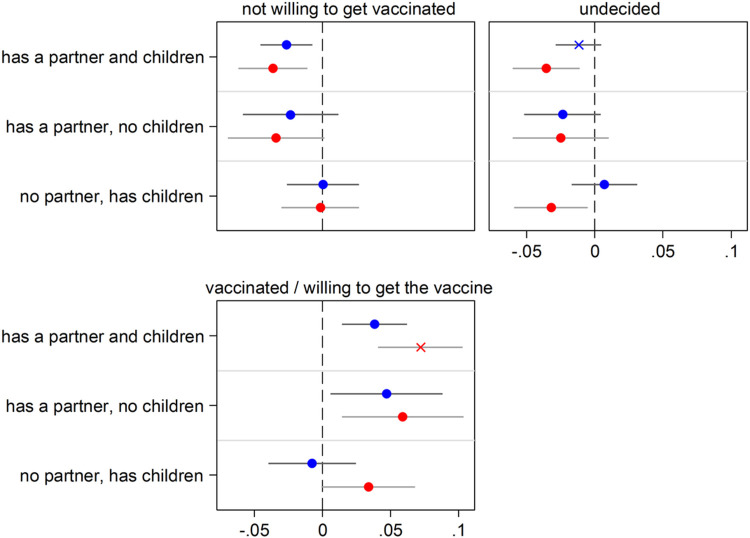
Having close kin (partner and children) and COVID-19 vaccine acceptance by gender (women in blue; men in red) Notes: The graph shows results for the effect of the explanatory variable (having kin) in the form of Average Marginal Effects (AMEs) with 95% confidence intervals from a multinomial logistic regression model for the three-level categorical outcome vaccine acceptance. Each model includes an interaction between the explanatory variable and a dummy variable for gender. Thus, separate AMEs by gender are obtained (women in blue; men in red). Each AME compares the predicted probability of a certain outcome category (e.g., being vaccinated or willing to get the vaccine) for each one of the three groups of older adults who have kin available (e.g., those who have both a partner and children) with that for the reference group (kinless, i.e. older adults who lack both partner and children). Statistically significant differences (p<0.05) between the AMEs of the two genders are indicated by an “x” in correspondence of the bigger AME. All control variables are included in the models. Full estimates are available in Table S.6 in the Supplementary Materials. Data are from SHARE Corona Survey 2 (June-August 2021).

**Figure 7:**
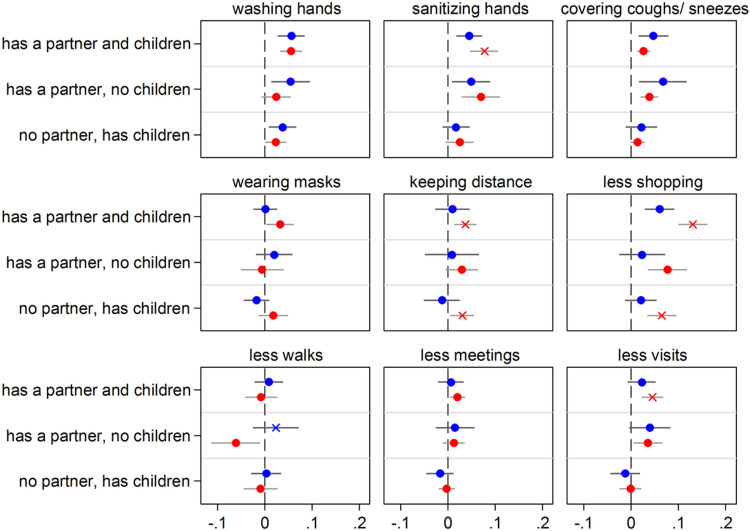
Having close kin (partner and children) and COVID-19 precautionary behaviors by country groups (South-East Europe in blue; North-West Europe in red) Notes: The graph shows the effect of the explanatory variable (having kin) in the form of Average Marginal Effects (AMEs) with 95% confidence intervals from nine separate logistic regression models (one for each of the considered precautionary behaviors). Each model includes an interaction between the explanatory variable and a dummy variable for country that distinguishes two groups (South-East Europe in blue; North-West Europe in red). Each AME compares the predicted probability of adopting a precautionary behavior for each one of the three groups of older adults who have kin available (e.g., those who have both a partner and children) with that for the reference group (kinless, i.e. older adults who lack both partner and children). Statistically significant differences (p<0.05) between the AMEs of the two considered country groups are indicated by an “x” in correspondence of the bigger AME. All control variables are included in the models. Full estimates are available in Table S.7 in the Supplementary Materials. Data are from SHARE Corona Survey 1 (June-August 2020).

**Figure 8:**
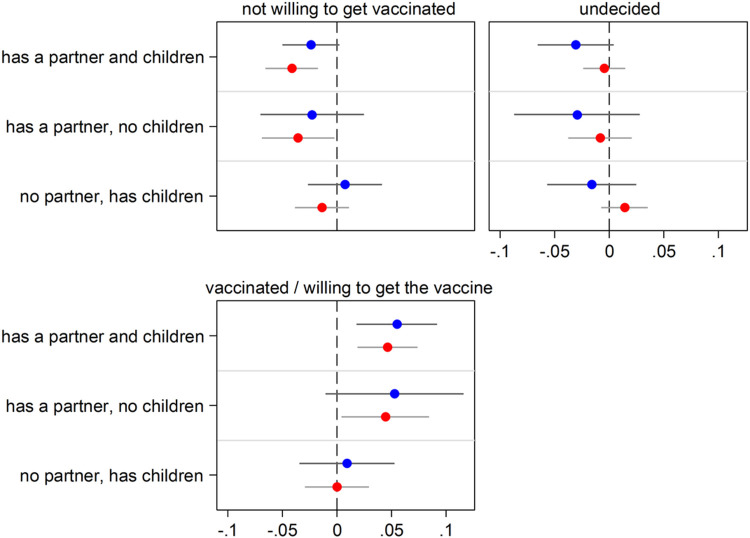
Having close kin (partner and children) and COVID-19 vaccine acceptance by country groups (South-East Europe in blue; North-West Europe in red) Notes: The graph shows results for the effect of the explanatory variable (having kin) in the form of Average Marginal Effects (AMEs) with 95% confidence intervals from a multinomial logistic regression model for the three-level categorical outcome vaccine acceptance. Each model includes an interaction between the explanatory variable and a dummy variable for country that distinguishes two groups (South-East Europe in blue; North-West Europe in red). Each AME compares the predicted probability of a certain outcome category (e.g., being vaccinated or willing to get the vaccine) for each one of the three groups of older adults who have kin available (e.g., those who have both a partner and children) with that for the reference group (kinless, i.e. older adults who lack both partner and children). Statistically significant differences (p<0.05) between the AMEs of the two considered country groups are indicated by an “x” in correspondence of the bigger AME. All control variables are included in the models. Full estimates are available in Table S.8 in the Supplementary Materials. Data are from SHARE Corona Survey 2 (June-August 2021).

Figures 3-8 present results obtained when interactions with gender, age groups, and country groups were considered to rule out the specificity of results for certain demographic or country groups (full regression estimates are available in Tables S.3-S.8 of the Supplementary Materials). Overall, results are very similar across gender (Figs. 3 and 4), age groups (Figs. 5 and 6) and country groups (Figs. 7 and 8), with statistically significant differences only observed in a bunch of cases, therefore confirming the importance of kinship (partnership, in particular) for precautionary behaviors and COVID-19 vaccine acceptance.

### Accounting for co-residence with kin

The stronger effect on precautionary behaviors and vaccine acceptance found for partnership as compared to parenthood might be driven by typical living arrangements with different kin at older ages. In our sample, the vast majority (96.2%) of partnered older adults live with their partner. Instead, only 16.3% of older parents co-reside with at least one of their children. Thus, partners might be more likely to provide support and to exert control as compared to children simply because of the higher amount of time (and resources) shared. However, even analyses that account for living arrangements show that (co-residing) partners more clearly influence precautionary behaviors and vaccine acceptance compared to (co-residing) children (see Figure S.1 for precautionary behaviors and Figure S.2 for vaccine acceptance in the Supplementary Materials).

### Additional analyses

As explained in the Methods section (see below), information on precautionary behaviors has been collected very differently in the second SHARE Corona Survey (SCS2) as compared to the first one. Nonetheless, analyses based on items in SCS2 yielded similar results to those based on items in SCS1: having close kin, and especially a partner, is associated with a higher probability of adopting precautionary behaviors (see Figures S.3 and S.4 in the Supplementary Material).

Finally, in additional preliminary analyses (available upon request) we also distinguished parents by their number of children but did not find this to matter. Also, given the slightly different sample size available for each outcome, we run the regression models selecting only observations available for all outcomes but results were barely affected.

## Discussion

Precautionary behaviors have demonstrated efficacy at containing the spread of COVID-19^56-58^. Similarly, COVID-19 vaccines have been found to reduce the risk of infection, hospitalization, and death^59-61^. Thus, to slow the spread of the Coronavirus and limit its negative health consequences it is crucial to understand the factors associated with individuals’ adoption of precautionary behaviors and acceptance of COVID-19 vaccines. Our study focuses on the role of kin ties among older people, which the general (pre-COVID) literature on health behaviors found to be crucial for the adoption of healthy behaviors^42-44,47-49,51-52^.

Our results show that having close kin is overall positively associated with older individuals’ likelihood of adopting precautionary behaviors and of being vaccinated or willing to get a COVID-19 vaccine. In particular, we find individuals in a partnership to be more likely to accept vaccine and to adopt (almost all) precautionary behaviors considered in this analysis. Results are robust to controlling for several other drivers of precautionary behaviors and vaccine acceptance (such as health and education), as well as to accounting for co-residence with kin. In addition, results are not specific for age, gender, or country groups. Most statistically significant associations are also substantially important. We find an adjusted difference in the probability of adoption of certain precautionary behavior (washing hand, using hand sanitizer, covering coughs and sneezes, reduced shopping) and of accepting COVID-19 vaccines of about 5 percentage points between partnered and unpartnered older adults, which is similar to the effect found in previous research for gender, health perception and chronic conditions^11,15,18,30-31^.

Although our data do not include direct measures of social control, the positive effect of kin on older people’s adoption of precautionary behaviors and vaccine acceptance in the context of the COVID-19 pandemic is in line with predictions from the pre-COVID literature which finds ample evidence of positive effects of family social control on health behaviors, such as avoidance of alcohol and cigarette consumption^38,62-64^. Thus, it can be speculated that during a pandemic partners and children have an important role in encouraging and controlling the respect of public health measures and recommendations to reduce the risk of contagion and its negative health effects. Evidence in our study is also consistent with social support mechanisms identified in pre-COVID studies, showing that motivational and practical help from close kin may positively influence health behaviors^38,54,65^. In the context of a pandemic, partners and children may provide assistance and useful information to understand the importance of precautionary behaviors and vaccination. Practical help may also be a mechanism at work. Indeed, among the health behaviors analyzed, we find that having children is especially important for a specific outcome, i.e., limiting in-person shopping. Children, in this case, might take the burden to go shop or order groceries online for their parents in order to reduce their risk of meeting strangers in a crowded indoor space, and therefore their risk of infection^66-68^.

The generally stronger role that we find for partners compared to children in influencing precautionary behaviors and vaccine acceptance also fits with the predominant evidence in the general literature on family and health behaviors that reports larger associations with health behaviors of being in a partnership than of having children^48,52-53^. This is in part explained by the stronger and more effective social control received by partners^38,63^, and by their usually greater provision of emotional and practical support^41,52,69^. In addition, partners have been found to bilaterally influence each other’s behaviors, thus reinforcing the social support and control function of being in a partnership^70^. In addition, previous studies found that concerns about the possible consequences of COVID-19 for family members influences precautionary behavior and vaccine acceptance^30^. This mechanism might also contribute explaining the stronger effect we find for partnership than for parenthood: older individuals might be more concerned about reducing the risk of infecting their partner than their children, because partners are more likely to be older individuals with health pre-conditions.

Our findings should be considered in light of some limitations. Our data could not account for the quality of relationships with partner and children for those individuals who have these ties. Previous research found that in case of conflicting relationships, family ties may also lead to health-compromising behaviors as coping mechanisms to deal with stress^71^. Also, the effectiveness of social control may vary with the type of behavior of the agent of the control^72^. Future research could examine more in detail possible heterogeneity in the role of kin ties in the context of the COVID-19 pandemic related to these and other factors (e.g., geographical distance to children and availability of friends). Also, an interesting avenue for future research is to examine the role of kin’s characteristics such as education and health. Finally, our results might be affected by differential response rates by family status during the pandemic. However, data quality controls showed that response rates remained satisfactory^73^.

Despite these limitations, our findings shed some light on the complex role of kin ties during the COVID-19 pandemic. It has been argued that family relationships (measured, for example, in terms of co-residence, frequent face-to-face contacts, etc.) may increase the chances of getting in contact with an infected person, thus constituting a risk to contract the virus. While it has been shown that conditional on having a (co-resident) family member infected the risk of getting the Coronavirus substantially increases^74-75^, the evidence on the (unconditional) risk of Coronavirus infection due to family ties *per se* is still scarce and, with few exceptions, is based on macro-level data. Also such macro-level analyses show mixed results^76-82^. A recent study^83^ based on part of the same individual-level data we used (SCS1) found that living with children was associated with a lower risk of Coronavirus infection for older women. Although it was not the focus on their study, the authors also found a similar effect for living with a partner for both men and women. These results are consistent with our findings of a positive association of close kin ties with precautionary behaviors and vaccine acceptance.

As Ross and colleagues^52^ wrote well before the onset of the COVID-19 pandemic, “a family is more than just a collection of people who might expose each other to infections and pollutants.” Thus, on the one hand, as all types of in-person contact, family contact can constitute a risk factor for Coronavirus infection. On the other hand, our study shows that partners, and to a lesser extent, children can also positively influence precautionary behaviors and vaccination. The overall effect of kin on risk of contagion and death is not easy to predict and it may vary with several factors, including extra-family (horizontal) relationships^84^ working status^85^ and age^86^ of family members. Our findings point to a potential positive role of kin in helping public health institutions to fight the pandemic and suggest that when analyzing the role of social relationships on COVID-19 outcomes rather than social network size *per se* one should account for (precautionary) behaviors and all types of contact (not limited to a specific type of ties, e.g., family) a person has. Understanding under which conditions social relationships may play a positive role in the context of a pandemic is of paramount importance and our study offers a new perspective and empirical evidence on this matter. Our findings that kin can have a positive influence on precautionary behaviors and vaccine acceptance suggest that policy makers and practitioners should focus especially on kinless individuals, especially those who are unpartnered, when designing measures to encourage the uptake and adherence to public health measures for COVID-19 prevention or in future pandemics.

## Methods

### Data

The present study used data from the Survey of Health, Ageing and Retirement in Europe (SHARE)^55^. SHARE is a longitudinal survey on non-institutionalized individuals aged 50 + and their partners in 27 European countries and Israel. It is conducted biannually since 2004 and 9 waves of data have been collected till date. We use data from wave 8, which started in October 2019 but was suspended in all countries in March 2020 due to the COVID-19 outbreak. Regular data collection is based on computer-assisted personal interviewing (CAPI), which provides pre-COVID information^87^. A special dataset, SHARE Corona Survey 1^88^, was added to wave 8. This survey has been administered with CATI (computer assisted telephone interviewing) between June and August 2020 to collect information on individuals’ behaviors and conditions during the pandemic (SHARE Corona Survey 1; SCS1). We excluded observations from Portugal (because Portugal started the fieldwork of the regular wave 8 only a few weeks before the start of the first lockdown due to the pandemic), thus restricting the analyses to individuals from the 27 countries included in both regular and SCS1 data. We also use data from wave 9, i.e. SHARE Corona Survey 2 (SCS2)^89^ collected between June and August 2021. Our outcome variables (precautionary behaviors and vaccine acceptance) come from the two SHARE Corona Surveys; independent variables, instead, are measured from the pre-COVID wave 8 of SHARE because these variables not available in the Corona Surveys. We dropped individuals older than 85 because they constituted a small share of the sample (about 4%) with almost no variation for certain outcomes. Results were however not affected by this selection. After discarding observations with missing values on the independent variables (640), our analytic samples comprise between 27,432 and 33,097 individuals depending on the outcome (the sample sizes differ because some outcomes do not apply to all respondents – those who declared who never left their home since the beginning of the pandemic – and because of missing values).

### Measures

Using data from SCS1 we built nine outcome variables corresponding to nine different precautionary health behaviors. The questionnaire of the SCS1 is available at: http://www.share-project.org/data-documentation/questionnaires/corona-questionnaire-1.html. All outcome variables are binary and coded so that 1 represents a precautionary behavior. More specifically the variables are constructed as follows (note that all of them refer to activities done or not since the outbreak of the pandemic and that italicized words refer to the names of the variables used in the models and reported in figures and tables). *Washing hands* equals one for respondents who report washing hands more than usual. *Sanitizing hands* equals one for respondents who report using special hand sanitizer or disinfection fluids more frequently than usual. *Covering coughs and sneezes* equals one for respondents who report paying special attention to covering cough and sneeze. *Wearing masks* equals one for respondents who report always wearing a face mask when in a public space. *Keeping distance* equals one for respondents who report always keeping distance from others in public. *Less shopping* equals one for respondents who report to have left their home for shopping less often or not at all since the outbreak of the pandemic. *Less walks* equals one for respondents who report to have left their home for going out for a walk less often or not at all since the outbreak of the pandemic. *Less meetings* equals one for respondents who report to have left their home for meeting with more than 5 people from outside their household less often or not at all since the outbreak of the pandemic. *Less visits* equals one for respondents who report to have left their home for visiting other family members less often or not at all since the outbreak of the pandemic.

The SCS2 used a different questionnaire (available at: http://www.share-project.org/data-documentation/questionnaires/corona-questionnaire-2.html), with questions which are not directly comparable with those in SCS1. Some of the questions about precautionary behaviors investigated in SCS1 were not kept in SCS2 (*Washing hands*; *Sanitizing hands; Wearing masks; Less walks; Less visits*); others changed the time reference (no longer ‘since the outbreak of the pandemic’ but either ‘in the three months preceding the survey’ or ‘compared to the first wave of the pandemic’); and there were some additional behaviors not included in SCS1 (such as going out to a restaurant). Therefore, these questions asked in SCS2 were only analyzed as robustness checks (and presented in Supplementary Files). Based on SCS2 items we built the following variables. *Infrequent shopping* equals one for respondents who report going out for shopping less often than once a week during the three months preceding the survey. *Infrequent meetings* equals one for respondents who report to have left their home for meeting with more than 5 people from outside their household less often than once a week during the three months preceding the survey. *More covering of cough/sneeze* equals one for respondents who report paying special attention to covering cough and sneeze more frequently. *Keeping distance* equals one for respondents who report to always pay special attention to keep distance from others in public during the three months preceding the survey. *Infrequent restaurants* equals one for respondents who report going out to a restaurant less often than once a week during the three months preceding the survey.

SCS2 additionally collected information in two consecutive steps on vaccination status and intent to get vaccinated. First, respondents were asked whether they had been vaccinated against COVID-19 at least once. Second, those who had not yet been vaccinated, were asked about their intention to do so, distinguishing whether they already had scheduled an appointment for vaccination, wanted to get vaccinated, did not want to get vaccinated, or were still undecided. We combined the information from these two questions and built a three-level categorical outcome variable: *vaccinated or willing to get the vaccine* (including vaccinated individuals and those who intend to get vaccinated); *undecided*; *not willing to get the vaccine*.

The explanatory variable combined information on partnership and parenthood status: *has a partner and children* (respondents who are in a partnership and have at least one child); *has a partner, no children* (childless respondents with a partner); *no partner, has children* (unpartnered respondents, including widowed or divorced respondents, with at least one child); *no partner, no children* (respondents with no close kin – reference category in the models). In the main analyses we do not distinguish our main independent variable of interest according to living arrangements, i.e. we only account for having kin independently of whether respondents live with their partner or child(ren). In a robustness check we built a similar variable but considering kin availability only in case of co-residence (i.e. we dropped respondents who did not live with their partner or at least one child). The resulting variable had the following categories: *has co-residing partner and children* (respondents who live with their partner and with at least one child); *has cores. partner, no children* (childless respondents with a co-resident partner); *no partner, has co-res. children* (unpartnered respondents with at least one co-resident child); *no partner, no children* (respondents with no close kin – reference category).

Control variables included the following: *Age* (in 5-year categories: *50–54* – reference category; *55–59*; *60–64*; *65–69*; *70–74*; *75–79*; *80–85*); *Female* (gender of respondent; *male* – reference category); *Education* (*low* – reference category; *medium*; *high*); *Working status* (*retired* – reference category; *working*; *other*); (Equivalized) *Household income* (continuous); *Self-rated health* (= 1 for respondents who rate their health as *fair* or *poor*, = 0 if health is rated as *good, very good* or *excellent*); *Diagnosed illness* (= 1 for respondents who self-reports of at least one doctor-diagnosed conditions including hypertension, diabetes, cancer, lung disease, heart disease, stroke and arthritis; = 0 otherwise); *Gali* (global activity limitations; = 1 for respondents whose activities are limited or severely limited because of health problems; = 0 otherwise); *Respondent or close relatives tested positive* (= 1 if the respondent or a close relative (partner, children, parents) has been tested for the Coronavirus and the result was positive; = 0 otherwise); *Country* of residence (reference category: *Austria*); *Week of interview*. As for education, the three groups are defined based on the International Standard Classification of Education (ISCED; http://www.uis.unesco.org/): low (ISCED 0–1, no or primary education, and ISCED 2, lower secondary education), medium (ISCED 3–4, higher secondary education), and high (ISCED 5–6, tertiary education). The independent variables have been measured using information from the regular SHARE wave 8 either because they are time-invariant characteristics of because of unavailability of the information in the two SHARE Corona Surveys.

### Analyses

Each of the precautionary health behavior described above represented a different binary outcome that we modeled using logistic regression. Thus, based on data from the SCS1 we estimated nine logistic regression models, one for each outcome. For vaccine acceptance, we use a multinomial logistic regression model. Although an ordering of the three categories of the outcome can be established (in terms of vaccine acceptance), a multinomial model allowed a higher degree of flexibility compared to an ordered logistic regression (i.e., it was possible to estimate separate effects of the independent variables for each category of the outcome).

To ease interpretation of results, the main findings are reported in the main text graphically as Average Marginal Effects (AMEs) for the explanatory variable with 95% confidence intervals. Due to the categorical nature of our outcomes and explanatory variables, the AMEs are to be interpreted as the discrete effect of the independent variable (compared to the reference category – *no partner, no children*), i.e. as the difference between the predicted probabilities (in percentage points) across the groups being compared (e.g., *has a partner and children* vs *no partner, no children*). Full tables of regression estimates (estimated coefficients; log-odds) are reported in the Supplementary Materials. All control variables listed above have been included in all regression models.

Among the additional analyses implemented, we considered heterogeneity analyses to rule out that the main findings only applied to certain demographic (gender and age) or country groups. More specifically, we have re-estimated the models that generated the main results by adding interactions between the explanatory variable and, in turn, gender, age (two groups: 50–64; 65+) and country groups. Countries have been grouped geographically: Northern/Central Europe (Denmark, Finland, France, Germany, Luxembourg, Netherlands, Sweden, Switzerland) and Southern/Eastern Europe (Bulgaria, Croatia, Cyprus, Czech Republic, Estonia, Greece, Hungary, Israel, Italy, Latvia, Lithuania, Malta, Poland, Romania, Slovakia, Slovenia, Spain). Although simple, this classification allows to capture considerable variation in family norms and similar grouping have been used in a previous study about the role of families on health and mortality^90^. Similarly to what we did for the main analyses, results are presented graphically, showing the estimated AMEs for the three categories of the explanatory variable corresponding to having close kin. However, this time we estimated separate AMEs for the two groups defined, in turn, by gender, age or country. In the few cases of a statistically significant difference (at the 5% level) between the AMEs this is indicated by an ‘*x*’ used as a marker (the ‘*x*’ is used for the highest AME among the two compared). Full tables of regression estimates (estimated coefficients; log-odds) are reported in the Supplementary Materials.

## Supplementary Material

Supplement 1
