# Supplementary material for: Close kin influence COVID-19 precautionary behaviors and vaccine acceptance of older individuals
Source: Res Sq. 2022 Jun 2:rs.3.rs-1699988. Preprint. [Version 1] doi: 10.21203/rs.3.rs-1699988/v1 (PMC9176653; doi:10.21203/rs.3.rs-1699988/v1)
Supplement: Supplement 1 [file 37c842e6e076bbdaa7ed929d.docx]

**Supplementary Materials: Additional Figures and Tables**

**Figure S.1 – Coresidence with close kin (partner and children) and COVID-19 precautionary behaviors**

Notes: The graph shows results (Average Marginal Effects (AMEs) with 95% confidence intervals) from nine separate logistic regression models for each of the considered precautionary behaviors. In particular, the effect of the explanatory variable (coresidence with kin) is presented in the form of AMEs. Each AME compares the predicted probability of adopting a precautionary behavior for each one of the three groups of older adults who have kin available and coreside with them (e.g., those who coreside with both a partner and at least one child) with that for the reference group (kinless, i.e. older adults who lack both partner and children). All control variables are included in the models. Data are from SHARE Corona Survey 1 (June-August 2020).

**Fig. S.2 – Coresidence with close kin (partner and children) and COVID-19 vaccine acceptance**

Notes: The graph shows results (Average Marginal Effects (AMEs) with 95% confidence intervals) from a multinomial logistic regression model for the three-level categorical outcome vaccine acceptance. In particular, the effect of the explanatory variable (coresidence with kin) is presented in the form of AMEs. Each AME compares the predicted probability of a certain outcome category (e.g., being vaccinated or willing to get the vaccine) for each one of the three groups of older adults who have kin available and coreside with them (e.g., those who coreside with both a partner and at least one child) with that for the reference group (kinless, i.e. older adults who lack both partner and children). All control variables are included in the models. Data are from SHARE Corona Survey 2 (June-August 2021).

**Fig. S.3 – Having close kin (partner and children) and COVID-19 precautionary behaviors in June-August 2021 (SHARE Corona Survey 2)**

Notes: The graph shows results (Average Marginal Effects (AMEs) with 95% confidence intervals) from five separate logistic regression models for each of the considered precautionary behaviors. In particular, the effect of the explanatory variable (kin availability) is presented in the form of AMEs. Each AME compares the predicted probability of adopting a precautionary behavior for each one of the three groups of older adults who have kin available (e.g., those who have both a partner and children) with that for the reference group (kinless, i.e. older adults who lack both partner and children). All control variables are included in the models. Data are from SHARE Corona Survey 2 (June-August 2021).

**Figure S.4 – Coresidence with close kin (partner and children) and COVID-19 precautionary behaviors in June-August 2021 (SHARE Corona Survey 2)**

Notes: The graph shows results (Average Marginal Effects (AMEs) with 95% confidence intervals) from nine separate logistic regression models for each of the considered precautionary behaviors. In particular, the effect of the explanatory variable (coresidence with kin) is presented in the form of AMEs. Each AME compares the predicted probability of adopting a precautionary behavior for each one of the three groups of older adults who have kin available and coreside with them (e.g., those who coreside with both a partner and at least one child) with that for the reference group (kinless, i.e. older adults who lack both partner and children). All control variables are included in the models. Data are from SHARE Corona Survey 2 (June-August 2021).

**Table S.1 – Logistic regression models for nine COVID-19 precautionary behaviors, full results**

| Independent variables | Outcome variables (COVID-19 precautionary behaviors) | | | | | | | | |
| --- | --- | --- | --- | --- | --- | --- | --- | --- | --- |
|  | washing | sanitizing | covering coughs | wearing | keeping | less | less | less | less |
|  | hands | hands | and sneezes | masks | distance | shopping | walks | meetings | visits |
| *Close kin availability (Ref.: no partner, no children)* | | | | | | | | | |
| has a partner and children | 0.54*** | 0.43*** | 0.34*** | 0.12 | 0.18** | 0.56*** | 0.01 | 0.18* | 0.31*** |
|  | (0.07) | (0.07) | (0.07) | (0.07) | (0.07) | (0.06) | (0.06) | (0.09) | (0.07) |
| has a partner, no children | 0.34** | 0.43*** | 0.52*** | 0.07 | 0.14 | 0.29** | -0.08 | 0.16 | 0.32** |
|  | (0.12) | (0.11) | (0.12) | (0.11) | (0.10) | (0.09) | (0.09) | (0.14) | (0.12) |
| no partner, has children | 0.28*** | 0.15* | 0.17* | -0.01 | 0.09 | 0.25*** | -0.01 | -0.09 | -0.05 |
|  | (0.08) | (0.07) | (0.07) | (0.08) | (0.07) | (0.06) | (0.06) | (0.09) | (0.08) |
| *Age (Ref.: 50-54)* |  |  |  |  |  |  |  |  |  |
| age 55-59 | 0.23* | 0.45*** | 0.47*** | -0.03 | 0.00 | -0.18* | -0.45*** | -0.09 | -0.26** |
|  | (0.11) | (0.09) | (0.10) | (0.10) | (0.08) | (0.08) | (0.08) | (0.10) | (0.09) |
| age 60-64 | 0.32*** | 0.45*** | 0.51*** | 0.08 | 0.05 | -0.16* | -0.40*** | -0.02 | -0.16 |
|  | (0.10) | (0.09) | (0.09) | (0.10) | (0.08) | (0.07) | (0.07) | (0.10) | (0.09) |
| age 65-69 | 0.38*** | 0.34*** | 0.36*** | 0.10 | 0.14 | -0.08 | -0.35*** | 0.02 | -0.01 |
|  | (0.10) | (0.08) | (0.09) | (0.10) | (0.08) | (0.08) | (0.07) | (0.10) | (0.09) |
| age 70-74 | 0.36*** | 0.27** | 0.25** | 0.18 | 0.24** | 0.12 | -0.14 | 0.24* | 0.23* |
|  | (0.10) | (0.08) | (0.09) | (0.10) | (0.08) | (0.08) | (0.08) | (0.10) | (0.09) |
| age 75-79 | 0.22* | 0.01 | 0.08 | 0.14 | 0.20* | 0.15 | 0.03 | 0.43*** | 0.30** |
|  | (0.10) | (0.09) | (0.09) | (0.10) | (0.09) | (0.08) | (0.08) | (0.11) | (0.10) |
| age 80-85 | 0.09 | -0.16 | -0.08 | 0.17 | 0.16 | 0.39*** | 0.27*** | 0.44*** | 0.39*** |
|  | (0.10) | (0.09) | (0.09) | (0.11) | (0.09) | (0.08) | (0.08) | (0.12) | (0.10) |
| Female | 0.28*** | 0.22*** | 0.27*** | 0.53*** | 0.40*** | 0.64*** | 0.32*** | 0.43*** | 0.33*** |
|  | (0.04) | (0.03) | (0.03) | (0.04) | (0.03) | (0.03) | (0.03) | (0.04) | (0.03) |
| *Education (Ref.: low)* |  |  |  |  |  |  |  |  |  |
| medium | 0.12 | 0.25*** | 0.19*** | 0.20** | 0.11 | 0.00 | -0.06 | 0.10 | -0.01 |
|  | (0.07) | (0.05) | (0.06) | (0.07) | (0.06) | (0.05) | (0.05) | (0.08) | (0.07) |
| high | 0.17** | 0.43*** | 0.23*** | 0.24*** | 0.18*** | -0.07 | -0.25*** | 0.08 | -0.13* |
|  | (0.06) | (0.05) | (0.05) | (0.05) | (0.05) | (0.05) | (0.04) | (0.07) | (0.06) |
| *Working status (Ref.: Retired)* | | | | | | | | | |
| working | 0.21** | 0.38*** | 0.24*** | -0.04 | -0.10* | -0.30*** | -0.15*** | -0.73*** | -0.15** |
|  | (0.07) | (0.06) | (0.06) | (0.06) | (0.05) | (0.04) | (0.04) | (0.06) | (0.05) |
| other | -0.07 | -0.09 | -0.06 | -0.03 | -0.02 | -0.02 | 0.18*** | -0.07 | -0.02 |
|  | (0.06) | (0.05) | (0.06) | (0.06) | (0.05) | (0.05) | (0.05) | (0.07) | (0.06) |
| Household income | 0.02* | -0.00 | 0.01 | -0.00 | 0.00 | 0.02* | -0.01* | 0.02 | 0.02* |
|  | (0.01) | (0.00) | (0.01) | (0.01) | (0.01) | (0.01) | (0.01) | (0.01) | (0.01) |
| Self-rated health | -0.07** | -0.10*** | -0.03 | -0.02 | -0.02 | 0.14*** | 0.30*** | 0.05* | 0.15*** |
|  | (0.02) | (0.02) | (0.02) | (0.02) | (0.02) | (0.02) | (0.02) | (0.02) | (0.02) |
| Diagnosed illness | -0.04 | 0.18*** | 0.10* | 0.22*** | 0.11** | 0.06 | 0.03 | 0.05 | 0.01 |
|  | (0.05) | (0.04) | (0.04) | (0.04) | (0.04) | (0.03) | (0.03) | (0.05) | (0.04) |
| Gali | -0.08 | -0.07 | -0.09* | -0.02 | 0.01 | 0.28*** | 0.22*** | 0.24*** | 0.13** |
|  | (0.05) | (0.04) | (0.04) | (0.04) | (0.04) | (0.03) | (0.03) | (0.05) | (0.04) |
| Respondent or close | 0.13 | 0.08 | 0.23** | -0.01 | 0.02 | 0.12* | -0.13* | 0.03 | 0.04 |
| relatives tested positive | (0.08) | (0.07) | (0.08) | (0.07) | (0.06) | (0.06) | (0.06) | (0.08) | (0.07) |
| *Country (Ref.: Austria)* |  |  |  |  |  |  |  |  |  |
| Germany | -0.64** | -0.64*** | -0.91*** | 0.32 | -0.45** | -0.85*** | -0.71*** | -0.19 | -0.64*** |
|  | (0.20) | (0.16) | (0.22) | (0.17) | (0.15) | (0.14) | (0.14) | (0.19) | (0.16) |
| Sweden | -0.24 | -0.07 | -1.34*** | -5.57*** | -0.78*** | -0.20 | -1.54*** | -0.82*** | -0.27 |
|  | (0.21) | (0.16) | (0.22) | (0.34) | (0.15) | (0.14) | (0.15) | (0.18) | (0.17) |
| Netherlands | 0.03 | -0.38* | -1.22*** | -6.05*** | -0.70*** | -0.62*** | -1.15*** | 0.04 | -0.19 |
|  | (0.25) | (0.18) | (0.24) | (0.60) | (0.17) | (0.15) | (0.17) | (0.23) | (0.19) |
| Spain | 0.67** | 1.06*** | -0.98*** | 1.26*** | 0.47* | 0.77*** | 1.97*** | 0.92*** | 1.02*** |
|  | (0.25) | (0.19) | (0.23) | (0.21) | (0.19) | (0.17) | (0.16) | (0.26) | (0.22) |
| Italy | 0.15 | 0.53** | -1.21*** | 1.80*** | 0.54** | 0.84*** | 2.78*** | 1.03*** | 0.92*** |
|  | (0.22) | (0.17) | (0.22) | (0.21) | (0.18) | (0.15) | (0.16) | (0.22) | (0.19) |
| France | -0.72*** | 0.25 | -1.10*** | -0.21 | -0.76*** | -0.20 | 0.66*** | -0.15 | -0.20 |
|  | (0.21) | (0.17) | (0.22) | (0.17) | (0.16) | (0.14) | (0.14) | (0.19) | (0.17) |
| Denmark | -0.20 | 1.63*** | -0.56* | -5.51*** | -0.11 | -0.94*** | -1.50*** | -0.66*** | -0.50** |
|  | (0.22) | (0.21) | (0.23) | (0.30) | (0.16) | (0.14) | (0.15) | (0.19) | (0.17) |
| Greece | -0.08 | 0.14 | -0.87*** | -1.23*** | -1.57*** | -0.02 | 1.46*** | 0.06 | -0.13 |
|  | (0.20) | (0.16) | (0.22) | (0.17) | (0.15) | (0.14) | (0.13) | (0.18) | (0.16) |
| Switzerland | -0.17 | 0.48** | -1.02*** | -2.85*** | -0.52** | 0.28 | -0.39** | 0.55* | 0.19 |
|  | (0.22) | (0.17) | (0.23) | (0.18) | (0.16) | (0.15) | (0.14) | (0.22) | (0.18) |
| Belgium | -0.20 | -0.01 | -1.29*** | -1.30*** | -0.22 | 0.02 | 0.01 | 0.76*** | 0.46* |
|  | (0.21) | (0.17) | (0.22) | (0.17) | (0.16) | (0.14) | (0.14) | (0.22) | (0.18) |
| Israel | -0.28 | -0.73*** | -1.54*** | 0.63** | -0.99*** | -0.06 | 0.98*** | -0.44* | 0.20 |
|  | (0.23) | (0.17) | (0.23) | (0.21) | (0.18) | (0.16) | (0.16) | (0.21) | (0.20) |
| Czech Republic | -0.83*** | 0.04 | -1.45*** | 1.80*** | -0.64*** | -0.59*** | 0.45*** | -0.47** | -0.66*** |
|  | (0.20) | (0.16) | (0.22) | (0.19) | (0.15) | (0.14) | (0.13) | (0.18) | (0.16) |
| Poland | -0.01 | 0.12 | -0.82*** | 1.17*** | -0.71*** | -0.21 | 0.60*** | -0.33 | -0.05 |
|  | (0.21) | (0.16) | (0.22) | (0.19) | (0.16) | (0.14) | (0.14) | (0.19) | (0.17) |
| Luxembourg | -0.76*** | 0.33 | -0.95*** | 1.76*** | 0.41* | 0.72*** | 0.13 | 1.46*** | 0.62** |
|  | (0.22) | (0.19) | (0.24) | (0.24) | (0.20) | (0.17) | (0.15) | (0.32) | (0.21) |
| Hungary | -0.37 | -0.13 | 0.24 | -0.13 | -0.82*** | 0.36* | 1.54*** | -0.67** | 0.62** |
|  | (0.24) | (0.19) | (0.31) | (0.20) | (0.19) | (0.17) | (0.17) | (0.22) | (0.23) |
| Slovenia | -0.39 | 1.00*** | -1.88*** | 0.78*** | -0.28 | 1.17*** | -0.20 | 0.75*** | 0.89*** |
|  | (0.20) | (0.17) | (0.21) | (0.18) | (0.16) | (0.15) | (0.13) | (0.21) | (0.18) |
| Estonia | -0.17 | 0.41* | -1.98*** | -2.29*** | -0.60*** | 0.08 | -0.10 | -0.80*** | 0.22 |
|  | (0.21) | (0.16) | (0.21) | (0.17) | (0.16) | (0.14) | (0.13) | (0.18) | (0.17) |
| Croatia | -0.19 | 0.32 | -0.78*** | -1.40*** | -1.01*** | 0.22 | 1.25*** | 0.32 | 0.47* |
|  | (0.22) | (0.17) | (0.23) | (0.18) | (0.17) | (0.15) | (0.15) | (0.22) | (0.19) |
| Lithuania | 0.18 | 0.47** | -1.20*** | 1.25*** | -0.27 | 0.62*** | 0.74*** | 0.02 | 0.53** |
|  | (0.23) | (0.18) | (0.23) | (0.20) | (0.17) | (0.16) | (0.14) | (0.21) | (0.19) |
| Bulgaria | -0.06 | -0.60*** | -1.25*** | 0.42* | -0.22 | -0.71*** | 1.15*** | -1.11*** | -0.78*** |
|  | (0.23) | (0.17) | (0.23) | (0.19) | (0.18) | (0.15) | (0.15) | (0.19) | (0.17) |
| Cyprus | 0.10 | 1.59*** | -2.52*** | -0.63** | -0.75*** | 0.86*** | 2.10*** | 0.39 | 0.57* |
|  | (0.27) | (0.28) | (0.23) | (0.20) | (0.20) | (0.21) | (0.19) | (0.28) | (0.25) |
| Finland | -0.02 | 0.71*** | -0.79*** | -5.11*** | -1.37*** | -0.31* | -1.19*** | -0.46* | -0.38* |
|  | (0.22) | (0.18) | (0.23) | (0.30) | (0.16) | (0.14) | (0.15) | (0.19) | (0.17) |
| Latvia | -0.25 | -0.24 | -1.41*** | -3.54*** | -1.68*** | -0.17 | 0.10 | -0.44* | 0.21 |
|  | (0.23) | (0.18) | (0.23) | (0.23) | (0.17) | (0.16) | (0.15) | (0.21) | (0.20) |
| Malta | 0.66* | 0.88*** | -0.89*** | -0.21 | 0.46* | 0.97*** | 1.86*** | 0.68** | 0.99*** |
|  | (0.27) | (0.20) | (0.24) | (0.20) | (0.23) | (0.18) | (0.16) | (0.25) | (0.23) |
| Romania | 0.05 | -0.11 | -1.00*** | 0.93*** | 0.03 | 0.61*** | 2.95*** | 0.31 | 0.65*** |
|  | (0.22) | (0.17) | (0.23) | (0.19) | (0.18) | (0.15) | (0.18) | (0.22) | (0.19) |
| Slovakia | -0.57** | -0.18 | -2.26*** | 0.24 | -1.38*** | -0.74*** | 0.55*** | -1.11*** | -0.63*** |
|  | (0.22) | (0.18) | (0.22) | (0.19) | (0.16) | (0.15) | (0.15) | (0.19) | (0.17) |
| Constant | 1.50*** | 0.49* | 2.10*** | 0.16 | 1.23*** | -0.31 | -1.02*** | 1.78*** | 0.92*** |
|  | (0.25) | (0.20) | (0.25) | (0.23) | (0.20) | (0.18) | (0.18) | (0.25) | (0.22) |
| N | 33097 | 33101 | 32926 | 27986 | 27968 | 32829 | 32606 | 32488 | 32504 |

Note: Estimated coefficients (effects on log-odds) from separate logistic regression models for each outcome. Standard errors in parentheses. *** p<0.001; *** p<0.01; *** p<0.05. Estimates correspond to the AMEs represented in Fig. 1 in the main manuscript. A set of dummy variables for the week of interview are also included but coefficients are not shown to save space. Data are from SHARE Corona Survey 1 (June-August 2020).

**Table S.2 – Multinomial logistic regression model for COVID-19 vaccine acceptance, full results**

| Independent variables | Outcome categories | |
| --- | --- | --- |
|  | (Ref.: vaccinated/willing to get the vaccine) | |
|  | not willing | undecided |
|  | to get vaccinated |  |
| *Close kin availability (Ref.: no partner, no children)* | | |
| has a partner and children | -0.48*** | -0.44*** |
|  | (0.10) | (0.11) |
| has a partner, no children | -0.44** | -0.44* |
|  | (0.17) | (0.18) |
| no partner, has children | -0.04 | -0.11 |
|  | (0.10) | (0.11) |
| *Age (Ref.: 50-54)* | | |
| age 55-59 | -0.18 | 0.07 |
|  | (0.12) | (0.12) |
| age 60-64 | -0.20 | -0.18 |
|  | (0.11) | (0.12) |
| age 65-69 | -0.31** | -0.29* |
|  | (0.12) | (0.12) |
| age 70-74 | -0.49*** | -0.61*** |
|  | (0.12) | (0.13) |
| age 75-79 | -0.56*** | -0.90*** |
|  | (0.13) | (0.14) |
| age 80-85 | -0.46*** | -1.08*** |
|  | (0.13) | (0.15) |
| Female | -0.04 | -0.08 |
|  | (0.05) | (0.05) |
| *Education (Ref.: low)* | | |
| medium | -0.11 | -0.01 |
|  | (0.09) | (0.10) |
| high | -0.39*** | -0.28** |
|  | (0.08) | (0.09) |
| *Working status (Ref.: Retired)* |  |  |
| working | 0.21** | 0.06 |
|  | (0.08) | (0.08) |
| other | 0.56*** | 0.32*** |
|  | (0.07) | (0.08) |
| Household income | -0.07*** | -0.15*** |
|  | (0.02) | (0.03) |
| Self-rated health | 0.12*** | 0.08* |
|  | (0.03) | (0.03) |
| Diagnosed illness | -0.17** | -0.22*** |
|  | (0.06) | (0.06) |
| Gali | 0.15* | 0.08 |
|  | (0.06) | (0.07) |
| Respondent or close relatives tested positive | -0.28*** | -0.10 |
|  | (0.05) | (0.05) |
| *Country (Ref.: Austria)* | | |
| Germany | -0.46* | -0.92*** |
|  | (0.18) | (0.27) |
| Sweden | -1.72*** | -1.62*** |
|  | (0.38) | (0.48) |
| Netherlands | -1.29*** | -1.71** |
|  | (0.37) | (0.61) |
| Spain | -2.04*** | -3.83*** |
|  | (0.33) | (1.02) |
| Italy | -1.15*** | -0.76** |
|  | (0.20) | (0.24) |
| France | -0.26 | -0.08 |
|  | (0.19) | (0.24) |
| Denmark | -1.80*** | -2.95*** |
|  | (0.32) | (0.73) |
| Greece | -0.34* | 0.49* |
|  | (0.17) | (0.20) |
| Switzerland | 0.74*** | 1.13*** |
|  | (0.18) | (0.23) |
| Belgium | -1.15*** | -1.55*** |
|  | (0.23) | (0.35) |
| Israel | -1.36*** | -1.30** |
|  | (0.34) | (0.49) |
| Czech Republic | -0.04 | 0.17 |
|  | (0.18) | (0.22) |
| Poland | 0.36* | 0.70*** |
|  | (0.17) | (0.21) |
| Luxembourg | -0.50 | -1.20** |
|  | (0.26) | (0.45) |
| Hungary | 0.20 | -0.74 |
|  | (0.23) | (0.39) |
| Slovenia | 0.58*** | 1.19*** |
|  | (0.17) | (0.20) |
| Estonia | 0.21 | 0.76*** |
|  | (0.16) | (0.20) |
| Croatia | 0.39* | 1.16*** |
|  | (0.18) | (0.21) |
| Lithuania | 0.86*** | 0.98*** |
|  | (0.18) | (0.23) |
| Bulgaria | 2.71*** | 3.21*** |
|  | (0.19) | (0.23) |
| Cyprus | -0.48 | 0.49 |
|  | (0.31) | (0.30) |
| Finland | -1.43*** | -0.94** |
|  | (0.28) | (0.32) |
| Latvia | 1.48*** | 1.90*** |
|  | (0.18) | (0.22) |
| Malta | -1.91*** | -2.92*** |
|  | (0.37) | (0.73) |
| Romania | 2.24*** | 2.24*** |
|  | (0.17) | (0.21) |
| Slovakia | 0.74*** | 0.91*** |
|  | (0.18) | (0.22) |
| Constant | -1.67*** | -2.06*** |
|  | (0.24) | (0.29) |
| N | 27432 | |

Estimated coefficients (effects on log-odds) from a multinomial logistic regression model for the three-level categorical outcome vaccine acceptance (reference = vaccinated/willing to get the vaccine). Standard errors in parentheses. *** p<0.001; *** p<0.01; *** p<0.05. Estimates correspond to the AMEs represented in Fig. 2 in the main manuscript. A set of dummy variables for the week of interview are also included but coefficients are not shown to save space. Data are from SHARE Corona Survey 2 (June-August 2021).

**Table S.3 – Logistic regression models for nine COVID-19 precautionary behaviors that include an interaction between the explanatory variable (kin availability) and age groups, full results**

| Independent variables | Outcome variables (COVID-19 precautionary behaviors) | | | | | | | | |
| --- | --- | --- | --- | --- | --- | --- | --- | --- | --- |
|  | washing | sanitizing | covering coughs | wearing | keeping | less | less | less | less |
|  | hands | hands | and sneezes | masks | distance | shopping | walks | meetings | visits |
| *Close kin availability (Ref.: no partner, no children)* | | | | | | | | | |
| has a partner and children | 0.52*** | 0.37** | 0.23 | 0.03 | 0.17 | 0.51*** | 0.04 | 0.11 | 0.26* |
|  | (0.13) | (0.12) | (0.13) | (0.12) | (0.10) | (0.09) | (0.10) | (0.12) | (0.11) |
| has a partner, no children | 0.35 | 0.36 | 0.58* | -0.21 | 0.06 | 0.34* | -0.15 | -0.01 | 0.11 |
|  | (0.21) | (0.20) | (0.23) | (0.18) | (0.16) | (0.14) | (0.15) | (0.19) | (0.17) |
| no partner, has children | 0.37** | 0.14 | 0.28* | -0.13 | 0.11 | 0.26** | -0.02 | -0.08 | 0.06 |
|  | (0.14) | (0.13) | (0.14) | (0.13) | (0.11) | (0.10) | (0.11) | (0.13) | (0.12) |
| 65 or older (ref.: younger than 65) | -0.62** | -1.12*** | -1.16*** | -0.41 | -0.18 | 0.97*** | 0.97*** | 0.90** | 0.65** |
|  | (0.24) | (0.22) | (0.22) | (0.23) | (0.20) | (0.19) | (0.19) | (0.28) | (0.23) |
| *Interactions between "65 or older" and:* | | | | | | | | | |
| has a partner and children | 0.03 | 0.09 | 0.14 | 0.14 | 0.00 | 0.09 | -0.04 | 0.14 | 0.08 |
|  | (0.15) | (0.14) | (0.15) | (0.15) | (0.13) | (0.12) | (0.13) | (0.17) | (0.15) |
| has a partner, no children | -0.04 | 0.08 | -0.10 | 0.47* | 0.14 | -0.07 | 0.13 | 0.37 | 0.44 |
|  | (0.25) | (0.24) | (0.27) | (0.23) | (0.21) | (0.18) | (0.19) | (0.28) | (0.24) |
| no partner, has children | -0.10 | 0.04 | -0.11 | 0.19 | -0.03 | -0.03 | -0.01 | -0.02 | -0.16 |
|  | (0.17) | (0.16) | (0.16) | (0.16) | (0.14) | (0.13) | (0.13) | (0.18) | (0.16) |
| *Age (ref.: 50-54)* |  |  |  |  |  |  |  |  |  |
| age 55-59 | -0.11 | -0.11 | -0.11 | -0.12 | -0.06 | 0.15 | -0.08 | 0.15 | -0.05 |
|  | (0.15) | (0.14) | (0.14) | (0.12) | (0.10) | (0.09) | (0.10) | (0.11) | (0.11) |
| age 60-64 | -0.04 | -0.15 | -0.11 | -0.02 | -0.02 | 0.20* | -0.00 | 0.24* | 0.06 |
|  | (0.15) | (0.14) | (0.14) | (0.12) | (0.10) | (0.09) | (0.10) | (0.11) | (0.11) |
| age 65-69 | 0.63*** | 0.76*** | 0.80*** | 0.25 | 0.24 | -0.71*** | -0.86*** | -0.69*** | -0.45** |
|  | (0.12) | (0.10) | (0.10) | (0.15) | (0.13) | (0.13) | (0.11) | (0.20) | (0.15) |
| age 70-74 | 0.60*** | 0.68*** | 0.68*** | 0.33* | 0.34** | -0.50*** | -0.65*** | -0.45* | -0.19 |
|  | (0.12) | (0.10) | (0.10) | (0.15) | (0.13) | (0.13) | (0.11) | (0.20) | (0.16) |
| age 75-79 | 0.46*** | 0.42*** | 0.50*** | 0.29 | 0.31* | -0.45*** | -0.48*** | -0.25 | -0.12 |
|  | (0.12) | (0.10) | (0.10) | (0.15) | (0.13) | (0.13) | (0.11) | (0.21) | (0.16) |
| age 80-85 | 0.32** | 0.24* | 0.34** | 0.31* | 0.26* | -0.21 | -0.23 | -0.23 | -0.01 |
|  | (0.12) | (0.10) | (0.11) | (0.15) | (0.13) | (0.13) | (0.12) | (0.21) | (0.16) |
| Female | 0.28*** | 0.21*** | 0.27*** | 0.53*** | 0.40*** | 0.66*** | 0.32*** | 0.45*** | 0.34*** |
|  | (0.04) | (0.03) | (0.03) | (0.04) | (0.03) | (0.03) | (0.03) | (0.04) | (0.03) |
| *Education (Ref.: low)* |  |  |  |  |  |  |  |  |  |
| medium | 0.11 | 0.23*** | 0.18** | 0.19** | 0.10 | 0.02 | -0.05 | 0.12 | -0.00 |
|  | (0.07) | (0.05) | (0.06) | (0.07) | (0.06) | (0.05) | (0.05) | (0.08) | (0.07) |
| high | 0.15** | 0.40*** | 0.21*** | 0.24*** | 0.18*** | -0.05 | -0.23*** | 0.11 | -0.11 |
|  | (0.06) | (0.05) | (0.05) | (0.06) | (0.05) | (0.05) | (0.04) | (0.07) | (0.06) |
| *Working status (Ref.: Retired)* |  |  |  |  |  |  |  |  |  |
| working | 0.15* | 0.28*** | 0.14* | -0.05 | -0.11* | -0.22*** | -0.08 | -0.66*** | -0.10 |
|  | (0.07) | (0.06) | (0.06) | (0.06) | (0.05) | (0.05) | (0.05) | (0.06) | (0.05) |
| other | -0.11 | -0.14** | -0.12* | -0.04 | -0.03 | 0.03 | 0.22*** | -0.02 | 0.01 |
|  | (0.06) | (0.05) | (0.06) | (0.06) | (0.05) | (0.05) | (0.05) | (0.08) | (0.06) |
| Household income | 0.02* | -0.00 | 0.01 | -0.00 | 0.00 | 0.02* | -0.01* | 0.02 | 0.02* |
|  | (0.01) | (0.00) | (0.01) | (0.01) | (0.01) | (0.01) | (0.01) | (0.01) | (0.01) |
| Self-rated health | -0.07** | -0.09*** | -0.03 | -0.02 | -0.02 | 0.14*** | 0.30*** | 0.05 | 0.15*** |
|  | (0.02) | (0.02) | (0.02) | (0.02) | (0.02) | (0.02) | (0.02) | (0.02) | (0.02) |
| Diagnosed illness | -0.04 | 0.18*** | 0.10* | 0.22*** | 0.11** | 0.06 | 0.03 | 0.05 | 0.01 |
|  | (0.05) | (0.04) | (0.04) | (0.04) | (0.04) | (0.03) | (0.03) | (0.05) | (0.04) |
| Gali | -0.08 | -0.06 | -0.09* | -0.02 | 0.01 | 0.27*** | 0.21*** | 0.23*** | 0.13** |
|  | (0.05) | (0.04) | (0.04) | (0.04) | (0.04) | (0.03) | (0.03) | (0.05) | (0.04) |
| Respondent or close | 0.13 | 0.07 | 0.22** | -0.01 | 0.02 | 0.13* | -0.13* | 0.04 | 0.05 |
| relatives tested positive | (0.08) | (0.07) | (0.08) | (0.07) | (0.06) | (0.06) | (0.06) | (0.08) | (0.07) |
| *Country (Ref.: Austria)* |  |  |  |  |  |  |  |  |  |
| Germany | -0.64** | -0.64*** | -0.91*** | 0.32 | -0.45** | -0.85*** | -0.71*** | -0.19 | -0.64*** |
|  | (0.20) | (0.16) | (0.22) | (0.17) | (0.15) | (0.14) | (0.14) | (0.19) | (0.16) |
| Sweden | -0.23 | -0.06 | -1.34*** | -5.57*** | -0.77*** | -0.21 | -1.54*** | -0.83*** | -0.27 |
|  | (0.21) | (0.16) | (0.22) | (0.34) | (0.15) | (0.14) | (0.15) | (0.18) | (0.17) |
| Netherlands | 0.04 | -0.37* | -1.21*** | -6.05*** | -0.70*** | -0.63*** | -1.16*** | 0.02 | -0.20 |
|  | (0.25) | (0.18) | (0.24) | (0.60) | (0.17) | (0.15) | (0.17) | (0.23) | (0.19) |
| Spain | 0.68** | 1.08*** | -0.96*** | 1.27*** | 0.47* | 0.76*** | 1.96*** | 0.90*** | 1.01*** |
|  | (0.25) | (0.19) | (0.23) | (0.21) | (0.19) | (0.17) | (0.16) | (0.26) | (0.22) |
| Italy | 0.15 | 0.53** | -1.21*** | 1.80*** | 0.54** | 0.84*** | 2.79*** | 1.04*** | 0.92*** |
|  | (0.22) | (0.17) | (0.22) | (0.21) | (0.18) | (0.15) | (0.16) | (0.22) | (0.19) |
| France | -0.72*** | 0.24 | -1.10*** | -0.21 | -0.76*** | -0.19 | 0.67*** | -0.14 | -0.20 |
|  | (0.21) | (0.17) | (0.22) | (0.17) | (0.16) | (0.14) | (0.14) | (0.19) | (0.17) |
| Denmark | -0.20 | 1.64*** | -0.56* | -5.51*** | -0.11 | -0.95*** | -1.51*** | -0.66*** | -0.50** |
|  | (0.22) | (0.21) | (0.23) | (0.30) | (0.16) | (0.14) | (0.15) | (0.19) | (0.17) |
| Greece | -0.08 | 0.15 | -0.86*** | -1.23*** | -1.57*** | -0.02 | 1.46*** | 0.06 | -0.12 |
|  | (0.20) | (0.16) | (0.22) | (0.17) | (0.15) | (0.14) | (0.13) | (0.19) | (0.16) |
| Switzerland | -0.16 | 0.49** | -1.01*** | -2.86*** | -0.52** | 0.27 | -0.40** | 0.54* | 0.18 |
|  | (0.22) | (0.17) | (0.23) | (0.18) | (0.16) | (0.15) | (0.14) | (0.22) | (0.18) |
| Belgium | -0.20 | -0.02 | -1.30*** | -1.30*** | -0.22 | 0.02 | 0.01 | 0.77*** | 0.46** |
|  | (0.21) | (0.17) | (0.22) | (0.17) | (0.16) | (0.14) | (0.14) | (0.22) | (0.18) |
| Israel | -0.27 | -0.70*** | -1.51*** | 0.64** | -0.98*** | -0.09 | 0.95*** | -0.47* | 0.19 |
|  | (0.23) | (0.17) | (0.23) | (0.21) | (0.18) | (0.16) | (0.16) | (0.21) | (0.20) |
| Czech Republic | -0.83*** | 0.03 | -1.45*** | 1.80*** | -0.64*** | -0.59*** | 0.45*** | -0.47* | -0.65*** |
|  | (0.20) | (0.16) | (0.22) | (0.19) | (0.15) | (0.14) | (0.13) | (0.18) | (0.16) |
| Poland | -0.02 | 0.09 | -0.84*** | 1.16*** | -0.71*** | -0.18 | 0.62*** | -0.30 | -0.02 |
|  | (0.21) | (0.16) | (0.22) | (0.19) | (0.16) | (0.14) | (0.14) | (0.19) | (0.17) |
| Luxembourg | -0.76*** | 0.31 | -0.96*** | 1.76*** | 0.40* | 0.74*** | 0.14 | 1.48*** | 0.64** |
|  | (0.22) | (0.19) | (0.24) | (0.24) | (0.20) | (0.17) | (0.15) | (0.32) | (0.21) |
| Hungary | -0.37 | -0.13 | 0.24 | -0.13 | -0.82*** | 0.36* | 1.55*** | -0.66** | 0.63** |
|  | (0.24) | (0.19) | (0.31) | (0.20) | (0.19) | (0.17) | (0.17) | (0.22) | (0.23) |
| Slovenia | -0.39 | 1.00*** | -1.88*** | 0.78*** | -0.28 | 1.18*** | -0.20 | 0.75*** | 0.89*** |
|  | (0.20) | (0.17) | (0.21) | (0.18) | (0.16) | (0.15) | (0.13) | (0.21) | (0.18) |
| Estonia | -0.17 | 0.41* | -1.97*** | -2.29*** | -0.60*** | 0.07 | -0.10 | -0.80*** | 0.21 |
|  | (0.21) | (0.16) | (0.21) | (0.17) | (0.16) | (0.14) | (0.13) | (0.18) | (0.17) |
| Croatia | -0.20 | 0.30 | -0.79*** | -1.40*** | -1.01*** | 0.24 | 1.27*** | 0.33 | 0.49** |
|  | (0.22) | (0.17) | (0.23) | (0.18) | (0.17) | (0.15) | (0.15) | (0.22) | (0.19) |
| Lithuania | 0.17 | 0.46* | -1.21*** | 1.25*** | -0.27 | 0.63*** | 0.76*** | 0.04 | 0.54** |
|  | (0.23) | (0.18) | (0.23) | (0.20) | (0.17) | (0.16) | (0.14) | (0.21) | (0.19) |
| Bulgaria | -0.06 | -0.61*** | -1.25*** | 0.41* | -0.22 | -0.70*** | 1.16*** | -1.10*** | -0.76*** |
|  | (0.23) | (0.17) | (0.23) | (0.19) | (0.18) | (0.15) | (0.15) | (0.19) | (0.17) |
| Cyprus | 0.09 | 1.58*** | -2.53*** | -0.63** | -0.75*** | 0.86*** | 2.10*** | 0.38 | 0.56* |
|  | (0.27) | (0.28) | (0.23) | (0.20) | (0.20) | (0.21) | (0.19) | (0.28) | (0.25) |
| Finland | -0.04 | 0.68*** | -0.83*** | -5.12*** | -1.37*** | -0.29* | -1.17*** | -0.44* | -0.37* |
|  | (0.22) | (0.18) | (0.23) | (0.30) | (0.16) | (0.14) | (0.15) | (0.19) | (0.17) |
| Latvia | -0.27 | -0.27 | -1.43*** | -3.54*** | -1.68*** | -0.15 | 0.12 | -0.42* | 0.23 |
|  | (0.23) | (0.18) | (0.23) | (0.23) | (0.17) | (0.16) | (0.15) | (0.21) | (0.20) |
| Malta | 0.66* | 0.88*** | -0.89*** | -0.21 | 0.46* | 0.98*** | 1.87*** | 0.69** | 1.00*** |
|  | (0.27) | (0.20) | (0.24) | (0.20) | (0.23) | (0.18) | (0.16) | (0.25) | (0.23) |
| Romania | 0.03 | -0.15 | -1.03*** | 0.92*** | 0.02 | 0.64*** | 2.98*** | 0.35 | 0.68*** |
|  | (0.22) | (0.17) | (0.23) | (0.19) | (0.18) | (0.16) | (0.18) | (0.22) | (0.19) |
| Slovakia | -0.60** | -0.24 | -2.32*** | 0.24 | -1.39*** | -0.69*** | 0.60*** | -1.06*** | -0.59*** |
|  | (0.22) | (0.18) | (0.22) | (0.19) | (0.16) | (0.15) | (0.15) | (0.19) | (0.17) |
| Constant | 1.89*** | 1.19*** | 2.82*** | 0.36 | 1.31*** | -0.70*** | -1.48*** | 1.50*** | 0.67** |
|  | (0.29) | (0.25) | (0.29) | (0.26) | (0.22) | (0.20) | (0.20) | (0.27) | (0.24) |
| N | 33097 | 33101 | 32926 | 27986 | 27968 | 32829 | 32606 | 32488 | 32504 |

Note: Estimated coefficients (effects on log-odds) from separate logistic regression models for each outcome. Standard errors in parentheses. *** p<0.001; *** p<0.01; *** p<0.05. Estimates correspond to the AMEs represented in Fig. 3 in the main manuscript. A set of dummy variables for the week of interview are also included but coefficients are not shown to save space.

**Table S.4 – Multinomial logistic regression model for COVID-19 vaccine acceptance that includes an interaction between the explanatory variable (kin availability) and age groups, full results**

| Independent variables | Outcome categories | |
| --- | --- | --- |
|  | (Ref.: vaccinated/willing to get the vaccine) | |
|  | not willing | undecided |
|  | to get vaccinated |  |
| *Close kin availability (Ref.: no partner, no children)* | | |
| has a partner and children | -0.18 | -0.29 |
|  | (0.16) | (0.16) |
| has a partner, no children | -0.20 | -0.62* |
|  | (0.26) | (0.27) |
| no partner, has children | 0.33 | -0.10 |
|  | (0.17) | (0.17) |
| 65 or older (ref.: younger than 65) | -0.08 | -1.06** |
|  | (0.30) | (0.33) |
| *Interactions between "65 or older" and:* | | |
| has a partner and children | -0.49* | -0.26 |
|  | (0.21) | (0.22) |
| has a partner, no children | -0.41 | 0.35 |
|  | (0.34) | (0.37) |
| no partner, has children | -0.56** | 0.01 |
|  | (0.21) | (0.23) |
| *Age (ref.: 50-54)* | | |
| age 55-59 | -0.38** | -0.28* |
|  | (0.14) | (0.14) |
| age 60-64 | -0.42** | -0.57*** |
|  | (0.14) | (0.14) |
| age 65-69 | 0.02 | 0.50* |
|  | (0.18) | (0.23) |
| age 70-74 | -0.17 | 0.17 |
|  | (0.18) | (0.23) |
| age 75-79 | -0.24 | -0.14 |
|  | (0.19) | (0.23) |
| age 80-85 | -0.15 | -0.34 |
|  | (0.19) | (0.24) |
| Female | -0.05 | -0.11* |
|  | (0.05) | (0.05) |
| *Education (Ref.: low)* | | |
| medium | -0.12 | -0.05 |
|  | (0.09) | (0.10) |
| high | -0.41*** | -0.33*** |
|  | (0.08) | (0.09) |
| *Working status (Ref.: Retired)* |  |  |
| working | 0.16* | -0.04 |
|  | (0.08) | (0.08) |
| other | 0.53*** | 0.25** |
|  | (0.08) | (0.08) |
| Household income | -0.07*** | -0.15*** |
|  | (0.02) | (0.03) |
| Self-rated health | 0.13*** | 0.09** |
|  | (0.03) | (0.03) |
| Diagnosed illness | -0.17** | -0.21*** |
|  | (0.06) | (0.06) |
| Gali | 0.16* | 0.09 |
|  | (0.06) | (0.07) |
| Respondent or close | -0.28*** | -0.10 |
| relatives tested positive | (0.05) | (0.05) |
| *Country (Ref.: Austria)* | | |
| Germany | -0.45* | -0.92*** |
|  | (0.18) | (0.27) |
| Sweden | -1.72*** | -1.61*** |
|  | (0.38) | (0.48) |
| Netherlands | -1.29*** | -1.68** |
|  | (0.37) | (0.61) |
| Spain | -2.02*** | -3.79*** |
|  | (0.33) | (1.02) |
| Italy | -1.15*** | -0.76** |
|  | (0.20) | (0.24) |
| France | -0.27 | -0.08 |
|  | (0.19) | (0.24) |
| Denmark | -1.80*** | -2.94*** |
|  | (0.32) | (0.73) |
| Greece | -0.34* | 0.50* |
|  | (0.17) | (0.20) |
| Switzerland | 0.75*** | 1.14*** |
|  | (0.18) | (0.23) |
| Belgium | -1.15*** | -1.55*** |
|  | (0.23) | (0.36) |
| Israel | -1.34*** | -1.25* |
|  | (0.34) | (0.49) |
| Czech Republic | -0.04 | 0.17 |
|  | (0.18) | (0.22) |
| Poland | 0.35* | 0.67** |
|  | (0.17) | (0.21) |
| Luxembourg | -0.51* | -1.23** |
|  | (0.26) | (0.45) |
| Hungary | 0.19 | -0.75 |
|  | (0.23) | (0.39) |
| Slovenia | 0.58*** | 1.19*** |
|  | (0.17) | (0.20) |
| Estonia | 0.21 | 0.77*** |
|  | (0.16) | (0.20) |
| Croatia | 0.39* | 1.14*** |
|  | (0.18) | (0.21) |
| Lithuania | 0.85*** | 0.97*** |
|  | (0.18) | (0.23) |
| Bulgaria | 2.71*** | 3.20*** |
|  | (0.19) | (0.23) |
| Cyprus | -0.47 | 0.51 |
|  | (0.31) | (0.30) |
| Finland | -1.44*** | -0.96** |
|  | (0.28) | (0.32) |
| Latvia | 1.47*** | 1.87*** |
|  | (0.18) | (0.22) |
| Malta | -1.91*** | -2.93*** |
|  | (0.37) | (0.73) |
| Romania | 2.23*** | 2.20*** |
|  | (0.17) | (0.21) |
| Slovakia | 0.72*** | 0.86*** |
|  | (0.18) | (0.22) |
| Constant | -1.70*** | -1.70*** |
|  | (0.29) | (0.32) |
| N | 27432 | |

Estimated coefficients (effects on log-odds) from a multinomial logistic regression model for the three-level categorical outcome vaccine acceptance (reference = vaccinated/willing to get the vaccine). Standard errors in parentheses. *** p<0.001; *** p<0.01; *** p<0.05. Estimates correspond to the AMEs represented in Fig. 4 in the main manuscript. A set of dummy variables for the week of interview are also included but coefficients are not shown to save space. Data are from SHARE Corona Survey 2 (June-August 2021).

**Table S.5 – Logistic regression models for nine COVID-19 precautionary behaviors that include an interaction between the explanatory variable (kin availability) and gender, full results**

| Independent variables | Outcome variables (COVID-19 precautionary behaviors) | | | | | | | | |
| --- | --- | --- | --- | --- | --- | --- | --- | --- | --- |
|  | washing | sanitizing | covering coughs | wearing | keeping | less | less | less | less |
|  | hands | hands | and sneezes | masks | distance | shopping | walks | meetings | visits |
| *Close kin availability (Ref.: no partner, no children)* | | | | | | | | | |
| has a partner and children | 0.75*** | 0.66*** | 0.46*** | 0.10 | 0.26** | 0.67*** | 0.10 | 0.42*** | 0.48*** |
|  | (0.10) | (0.09) | (0.10) | (0.11) | (0.09) | (0.08) | (0.09) | (0.11) | (0.10) |
| has a partner, no children | 0.56*** | 0.57*** | 0.73*** | 0.05 | 0.23 | 0.33** | 0.00 | 0.39* | 0.42** |
|  | (0.16) | (0.15) | (0.17) | (0.16) | (0.14) | (0.13) | (0.14) | (0.18) | (0.16) |
| no partner, has children | 0.39*** | 0.29** | 0.28** | 0.08 | 0.14 | 0.07 | -0.08 | -0.05 | -0.02 |
|  | (0.11) | (0.10) | (0.11) | (0.12) | (0.10) | (0.09) | (0.10) | (0.12) | (0.11) |
| Female (Ref.: male) | 0.65*** | 0.59*** | 0.49*** | 0.54*** | 0.54*** | 0.73*** | 0.43*** | 0.88*** | 0.61*** |
|  | (0.14) | (0.13) | (0.13) | (0.14) | (0.13) | (0.11) | (0.12) | (0.17) | (0.14) |
| *Interactions between "female" and:* | | | | | | | | | |
| has a partner and children | -0.44** | -0.44*** | -0.23 | 0.03 | -0.17 | -0.22 | -0.17 | -0.57** | -0.37* |
|  | (0.15) | (0.13) | (0.14) | (0.15) | (0.13) | (0.12) | (0.12) | (0.18) | (0.15) |
| has a partner, no children | -0.47 | -0.27 | -0.43 | 0.03 | -0.18 | -0.10 | -0.15 | -0.53 | -0.21 |
|  | (0.24) | (0.22) | (0.24) | (0.23) | (0.21) | (0.18) | (0.19) | (0.28) | (0.24) |
| no partner, has children | -0.28 | -0.31* | -0.23 | -0.13 | -0.11 | 0.25* | 0.06 | -0.21 | -0.13 |
|  | (0.16) | (0.14) | (0.15) | (0.16) | (0.14) | (0.12) | (0.13) | (0.19) | (0.16) |
| *Age (Ref.: 50-54)* |  |  |  |  |  |  |  |  |  |
| age 55-59 | 0.24* | 0.46*** | 0.47*** | -0.02 | 0.01 | -0.18* | -0.45*** | -0.09 | -0.26** |
|  | (0.11) | (0.09) | (0.10) | (0.10) | (0.08) | (0.08) | (0.08) | (0.10) | (0.09) |
| age 60-64 | 0.33*** | 0.45*** | 0.51*** | 0.09 | 0.05 | -0.16* | -0.40*** | -0.02 | -0.16 |
|  | (0.10) | (0.09) | (0.09) | (0.10) | (0.08) | (0.07) | (0.07) | (0.10) | (0.09) |
| age 65-69 | 0.38*** | 0.34*** | 0.36*** | 0.11 | 0.13 | -0.09 | -0.35*** | 0.01 | -0.02 |
|  | (0.10) | (0.08) | (0.09) | (0.10) | (0.08) | (0.08) | (0.07) | (0.10) | (0.09) |
| age 70-74 | 0.36*** | 0.27** | 0.25** | 0.19 | 0.23** | 0.10 | -0.15* | 0.23* | 0.23* |
|  | (0.10) | (0.08) | (0.09) | (0.10) | (0.08) | (0.08) | (0.08) | (0.10) | (0.09) |
| age 75-79 | 0.21* | 0.01 | 0.08 | 0.15 | 0.20* | 0.13 | 0.02 | 0.41*** | 0.28** |
|  | (0.10) | (0.09) | (0.09) | (0.10) | (0.09) | (0.08) | (0.08) | (0.11) | (0.10) |
| age 80-85 | 0.08 | -0.17 | -0.08 | 0.18 | 0.15 | 0.36*** | 0.26** | 0.41*** | 0.38*** |
|  | (0.10) | (0.09) | (0.09) | (0.11) | (0.09) | (0.08) | (0.08) | (0.12) | (0.10) |
| *Education (Ref.: low)* | | | | | | | | | |
| medium | 0.12 | 0.25*** | 0.19*** | 0.20** | 0.11 | 0.00 | -0.06 | 0.10 | -0.01 |
|  | (0.07) | (0.05) | (0.06) | (0.07) | (0.06) | (0.05) | (0.05) | (0.08) | (0.07) |
| high | 0.16** | 0.42*** | 0.23*** | 0.24*** | 0.18*** | -0.07 | -0.25*** | 0.08 | -0.13* |
|  | (0.06) | (0.05) | (0.05) | (0.05) | (0.05) | (0.05) | (0.04) | (0.07) | (0.06) |
| *Working status (Ref.: Retired)* | | | | | | | | | |
| working | 0.21** | 0.38*** | 0.24*** | -0.04 | -0.10* | -0.30*** | -0.15*** | -0.74*** | -0.16** |
|  | (0.07) | (0.06) | (0.06) | (0.06) | (0.05) | (0.04) | (0.04) | (0.06) | (0.05) |
| other | -0.06 | -0.07 | -0.05 | -0.04 | -0.02 | -0.01 | 0.19*** | -0.05 | -0.01 |
|  | (0.06) | (0.05) | (0.06) | (0.06) | (0.05) | (0.05) | (0.05) | (0.07) | (0.06) |
| Household income | 0.02* | -0.00 | 0.01 | -0.00 | 0.00 | 0.02** | -0.01* | 0.02 | 0.02* |
|  | (0.01) | (0.00) | (0.01) | (0.01) | (0.01) | (0.01) | (0.01) | (0.01) | (0.01) |
| Self-rated health | -0.07** | -0.10*** | -0.03 | -0.02 | -0.02 | 0.14*** | 0.30*** | 0.05* | 0.15*** |
|  | (0.02) | (0.02) | (0.02) | (0.02) | (0.02) | (0.02) | (0.02) | (0.02) | (0.02) |
| Diagnosed illness | -0.04 | 0.18*** | 0.10* | 0.22*** | 0.11** | 0.06 | 0.03 | 0.04 | 0.01 |
|  | (0.05) | (0.04) | (0.04) | (0.04) | (0.04) | (0.03) | (0.03) | (0.05) | (0.04) |
| Gali | -0.08 | -0.07 | -0.09* | -0.02 | 0.01 | 0.28*** | 0.22*** | 0.24*** | 0.13** |
|  | (0.05) | (0.04) | (0.04) | (0.04) | (0.04) | (0.03) | (0.03) | (0.05) | (0.04) |
| Respondent or close | 0.13 | 0.08 | 0.23** | -0.01 | 0.02 | 0.12* | -0.13* | 0.03 | 0.04 |
| relatives tested positive | (0.08) | (0.07) | (0.08) | (0.07) | (0.06) | (0.06) | (0.06) | (0.08) | (0.07) |
| *Country (Ref.: Austria)* | | | | | | | | | |
| Germany | -0.62** | -0.63*** | -0.90*** | 0.31 | -0.44** | -0.84*** | -0.70*** | -0.17 | -0.63*** |
|  | (0.20) | (0.16) | (0.22) | (0.17) | (0.15) | (0.14) | (0.14) | (0.19) | (0.16) |
| Sweden | -0.22 | -0.06 | -1.34*** | -5.58*** | -0.77*** | -0.18 | -1.52*** | -0.80*** | -0.25 |
|  | (0.21) | (0.16) | (0.22) | (0.34) | (0.15) | (0.14) | (0.15) | (0.18) | (0.17) |
| Netherlands | 0.04 | -0.37* | -1.22*** | -6.06*** | -0.70*** | -0.60*** | -1.14*** | 0.05 | -0.18 |
|  | (0.25) | (0.18) | (0.24) | (0.60) | (0.17) | (0.15) | (0.17) | (0.23) | (0.19) |
| Spain | 0.67** | 1.07*** | -0.98*** | 1.26*** | 0.47* | 0.77*** | 1.97*** | 0.92*** | 1.02*** |
|  | (0.25) | (0.19) | (0.23) | (0.21) | (0.19) | (0.17) | (0.16) | (0.26) | (0.22) |
| Italy | 0.16 | 0.53** | -1.20*** | 1.80*** | 0.54** | 0.84*** | 2.79*** | 1.04*** | 0.92*** |
|  | (0.22) | (0.17) | (0.22) | (0.21) | (0.18) | (0.15) | (0.16) | (0.22) | (0.19) |
| France | -0.71*** | 0.26 | -1.09*** | -0.22 | -0.76*** | -0.19 | 0.67*** | -0.13 | -0.19 |
|  | (0.21) | (0.17) | (0.22) | (0.17) | (0.16) | (0.14) | (0.14) | (0.19) | (0.17) |
| Denmark | -0.19 | 1.65*** | -0.56* | -5.52*** | -0.11 | -0.93*** | -1.49*** | -0.63*** | -0.48** |
|  | (0.22) | (0.21) | (0.23) | (0.30) | (0.16) | (0.14) | (0.15) | (0.19) | (0.17) |
| Greece | -0.08 | 0.14 | -0.87*** | -1.23*** | -1.57*** | -0.01 | 1.46*** | 0.06 | -0.13 |
|  | (0.20) | (0.16) | (0.22) | (0.17) | (0.15) | (0.14) | (0.13) | (0.19) | (0.16) |
| Switzerland | -0.16 | 0.49** | -1.02*** | -2.86*** | -0.51** | 0.30* | -0.38** | 0.57** | 0.20 |
|  | (0.22) | (0.17) | (0.23) | (0.18) | (0.16) | (0.15) | (0.14) | (0.22) | (0.18) |
| Belgium | -0.19 | -0.00 | -1.29*** | -1.31*** | -0.21 | 0.03 | 0.02 | 0.78*** | 0.47** |
|  | (0.21) | (0.17) | (0.22) | (0.17) | (0.16) | (0.14) | (0.14) | (0.22) | (0.18) |
| Israel | -0.28 | -0.73*** | -1.54*** | 0.63** | -0.99*** | -0.05 | 0.98*** | -0.43* | 0.21 |
|  | (0.23) | (0.17) | (0.23) | (0.21) | (0.18) | (0.16) | (0.16) | (0.21) | (0.20) |
| Czech Republic | -0.82*** | 0.05 | -1.44*** | 1.79*** | -0.64*** | -0.58*** | 0.45*** | -0.46* | -0.65*** |
|  | (0.20) | (0.16) | (0.22) | (0.19) | (0.15) | (0.14) | (0.13) | (0.18) | (0.16) |
| Poland | 0.00 | 0.13 | -0.82*** | 1.16*** | -0.70*** | -0.20 | 0.61*** | -0.31 | -0.04 |
|  | (0.21) | (0.16) | (0.22) | (0.19) | (0.16) | (0.14) | (0.14) | (0.19) | (0.17) |
| Luxembourg | -0.75*** | 0.33 | -0.95*** | 1.76*** | 0.41* | 0.72*** | 0.13 | 1.46*** | 0.63** |
|  | (0.22) | (0.19) | (0.24) | (0.24) | (0.20) | (0.17) | (0.15) | (0.32) | (0.21) |
| Hungary | -0.37 | -0.12 | 0.24 | -0.13 | -0.82*** | 0.36* | 1.54*** | -0.66** | 0.62** |
|  | (0.24) | (0.19) | (0.31) | (0.20) | (0.19) | (0.17) | (0.17) | (0.22) | (0.23) |
| Slovenia | -0.38 | 1.01*** | -1.87*** | 0.77*** | -0.27 | 1.19*** | -0.20 | 0.76*** | 0.90*** |
|  | (0.20) | (0.17) | (0.21) | (0.18) | (0.16) | (0.15) | (0.13) | (0.21) | (0.18) |
| Estonia | -0.17 | 0.41* | -1.98*** | -2.29*** | -0.60*** | 0.08 | -0.10 | -0.79*** | 0.22 |
|  | (0.21) | (0.16) | (0.21) | (0.17) | (0.16) | (0.14) | (0.13) | (0.18) | (0.17) |
| Croatia | -0.18 | 0.33 | -0.78*** | -1.40*** | -1.00*** | 0.23 | 1.26*** | 0.33 | 0.48* |
|  | (0.22) | (0.17) | (0.23) | (0.18) | (0.17) | (0.15) | (0.15) | (0.22) | (0.19) |
| Lithuania | 0.18 | 0.47** | -1.20*** | 1.25*** | -0.27 | 0.62*** | 0.75*** | 0.03 | 0.53** |
|  | (0.23) | (0.18) | (0.23) | (0.20) | (0.17) | (0.16) | (0.14) | (0.21) | (0.19) |
| Bulgaria | -0.06 | -0.59*** | -1.24*** | 0.42* | -0.22 | -0.70*** | 1.15*** | -1.10*** | -0.77*** |
|  | (0.23) | (0.17) | (0.23) | (0.19) | (0.18) | (0.15) | (0.15) | (0.19) | (0.17) |
| Cyprus | 0.10 | 1.59*** | -2.52*** | -0.63** | -0.75*** | 0.87*** | 2.10*** | 0.39 | 0.57* |
|  | (0.27) | (0.28) | (0.23) | (0.20) | (0.20) | (0.21) | (0.19) | (0.28) | (0.25) |
| Finland | -0.01 | 0.72*** | -0.79*** | -5.12*** | -1.36*** | -0.30* | -1.18*** | -0.45* | -0.37* |
|  | (0.22) | (0.18) | (0.23) | (0.30) | (0.16) | (0.14) | (0.15) | (0.19) | (0.17) |
| Latvia | -0.25 | -0.24 | -1.40*** | -3.54*** | -1.68*** | -0.17 | 0.10 | -0.44* | 0.21 |
|  | (0.23) | (0.18) | (0.23) | (0.23) | (0.17) | (0.16) | (0.15) | (0.21) | (0.20) |
| Malta | 0.66* | 0.88*** | -0.89*** | -0.21 | 0.46* | 0.98*** | 1.86*** | 0.68** | 0.99*** |
|  | (0.27) | (0.20) | (0.24) | (0.20) | (0.23) | (0.18) | (0.16) | (0.25) | (0.23) |
| Romania | 0.06 | -0.11 | -1.00*** | 0.93*** | 0.03 | 0.61*** | 2.95*** | 0.32 | 0.66*** |
|  | (0.22) | (0.17) | (0.23) | (0.19) | (0.18) | (0.16) | (0.18) | (0.22) | (0.19) |
| Slovakia | -0.56* | -0.17 | -2.26*** | 0.24 | -1.38*** | -0.73*** | 0.56*** | -1.09*** | -0.62*** |
|  | (0.22) | (0.18) | (0.22) | (0.19) | (0.16) | (0.15) | (0.15) | (0.19) | (0.17) |
| Constant | 1.33*** | 0.30 | 1.98*** | 0.15 | 1.16*** | -0.36 | -1.08*** | 1.59*** | 0.79*** |
|  | (0.26) | (0.21) | (0.26) | (0.24) | (0.21) | (0.19) | (0.19) | (0.25) | (0.23) |
| N | 33097 | 33101 | 32926 | 27986 | 27968 | 32829 | 32606 | 32488 | 32504 |

Note: Estimated coefficients (effects on log-odds) from separate logistic regression models for each outcome. Standard errors in parentheses. *** p<0.001; *** p<0.01; *** p<0.05. Estimates correspond to the AMEs represented in Fig. 5 in the main manuscript. A set of dummy variables for the week of interview are also included but coefficients are not shown to save space.

**Table S.6** **– Multinomial logistic regression model for COVID-19 vaccine acceptance that includes an interaction between the explanatory variable (kin availability) and gender, full results**

| Independent variables | Outcome categories | |
| --- | --- | --- |
|  | (Ref.: vaccinated/willing to get the vaccine) | |
|  | not willing | undecided |
|  | to get vaccinated |  |
| *Close kin availability (Ref.: no partner, no children)* | | |
| has a partner and children | -0.58*** | -0.61*** |
|  | (0.15) | (0.15) |
| has a partner, no children | -0.51* | -0.43 |
|  | (0.24) | (0.24) |
| no partner, has children | -0.11 | -0.45* |
|  | (0.16) | (0.18) |
| Female (Ref.: male) | -0.19 | -0.43* |
|  | (0.19) | (0.21) |
| *Interactions between "female" and:* |  |  |
| has a partner and children | 0.18 | 0.33 |
|  | (0.20) | (0.22) |
| has a partner, no children | 0.13 | -0.06 |
|  | (0.33) | (0.37) |
| no partner, has children | 0.13 | 0.56* |
|  | (0.21) | (0.24) |
| *Age (Ref.: 50-54)* | | |
| age 55-59 | -0.18 | 0.07 |
|  | (0.12) | (0.12) |
| age 60-64 | -0.20 | -0.19 |
|  | (0.11) | (0.12) |
| age 65-69 | -0.30** | -0.30* |
|  | (0.12) | (0.12) |
| age 70-74 | -0.48*** | -0.61*** |
|  | (0.12) | (0.13) |
| age 75-79 | -0.55*** | -0.90*** |
|  | (0.13) | (0.14) |
| age 80-85 | -0.46*** | -1.08*** |
|  | (0.13) | (0.15) |
| *Education (Ref.: low)* | | |
| medium | -0.11 | -0.01 |
|  | (0.09) | (0.10) |
| high | -0.39*** | -0.27** |
|  | (0.08) | (0.09) |
| *Working status (Ref.: Retired)* | | |
| working | 0.21** | 0.06 |
|  | (0.08) | (0.08) |
| other | 0.55*** | 0.32*** |
|  | (0.07) | (0.08) |
| Household income | -0.07*** | -0.15*** |
|  | (0.02) | (0.03) |
| Self-rated health | 0.12*** | 0.08* |
|  | (0.03) | (0.03) |
| Diagnosed illness | -0.17** | -0.22*** |
|  | (0.06) | (0.06) |
| Gali | 0.15* | 0.08 |
|  | (0.06) | (0.07) |
| Respondent or close | -0.28*** | -0.10 |
| relatives tested positive | (0.05) | (0.05) |
| *Country (Ref.: Austria)* | | |
| Germany | -0.47* | -0.92*** |
|  | (0.18) | (0.27) |
| Sweden | -1.72*** | -1.61*** |
|  | (0.38) | (0.48) |
| Netherlands | -1.29*** | -1.70** |
|  | (0.37) | (0.61) |
| Spain | -2.04*** | -3.83*** |
|  | (0.33) | (1.02) |
| Italy | -1.15*** | -0.75** |
|  | (0.20) | (0.24) |
| France | -0.26 | -0.07 |
|  | (0.19) | (0.24) |
| Denmark | -1.81*** | -2.96*** |
|  | (0.32) | (0.73) |
| Greece | -0.34* | 0.49* |
|  | (0.17) | (0.20) |
| Switzerland | 0.74*** | 1.14*** |
|  | (0.18) | (0.23) |
| Belgium | -1.15*** | -1.55*** |
|  | (0.23) | (0.36) |
| Israel | -1.36*** | -1.30** |
|  | (0.34) | (0.49) |
| Czech Republic | -0.04 | 0.17 |
|  | (0.18) | (0.22) |
| Poland | 0.36* | 0.70*** |
|  | (0.17) | (0.21) |
| Luxembourg | -0.50 | -1.20** |
|  | (0.26) | (0.45) |
| Hungary | 0.19 | -0.74 |
|  | (0.23) | (0.39) |
| Slovenia | 0.58*** | 1.19*** |
|  | (0.17) | (0.20) |
| Estonia | 0.20 | 0.77*** |
|  | (0.16) | (0.20) |
| Croatia | 0.39* | 1.16*** |
|  | (0.18) | (0.21) |
| Lithuania | 0.86*** | 0.99*** |
|  | (0.18) | (0.23) |
| Bulgaria | 2.71*** | 3.21*** |
|  | (0.19) | (0.23) |
| Cyprus | -0.48 | 0.50 |
|  | (0.31) | (0.30) |
| Finland | -1.43*** | -0.93** |
|  | (0.28) | (0.32) |
| Latvia | 1.48*** | 1.90*** |
|  | (0.18) | (0.22) |
| Malta | -1.91*** | -2.92*** |
|  | (0.37) | (0.73) |
| Romania | 2.25*** | 2.24*** |
|  | (0.17) | (0.21) |
| Slovakia | 0.74*** | 0.90*** |
|  | (0.18) | (0.22) |
| Constant | -1.58*** | -1.89*** |
|  | (0.26) | (0.30) |
| N | 27432 | |

Estimated coefficients (effects on log-odds) from a multinomial logistic regression model for the three-level categorical outcome vaccine acceptance (reference = vaccinated/willing to get the vaccine). Standard errors in parentheses. *** p<0.001; *** p<0.01; *** p<0.05. Estimates correspond to the AMEs represented in Fig. 6 in the main manuscript. A set of dummy variables for the week of interview are also included but coefficients are not shown to save space. Data are from SHARE Corona Survey 2 (June-August 2021).

**Table S.7 – Logistic regression models for nine COVID-19 precautionary behaviors that include an interaction between the explanatory variable (kin availability) and country groups, full results**

| Independent variables | Outcome variables (COVID-19 precautionary behaviors) | | | | | | | | |
| --- | --- | --- | --- | --- | --- | --- | --- | --- | --- |
|  | washing | sanitizing | covering coughs | wearing | keeping | less | less | less | less |
|  | hands | hands | and sneezes | masks | distance | shopping | walks | meetings | visits |
| *Close kin availability (Ref.: no partner, no children)* | | | | | | | | | |
| has a partner and children | 0.67*** | 0.59*** | 0.46*** | 0.24* | 0.30*** | 0.83*** | -0.04 | 0.35** | 0.46*** |
|  | (0.11) | (0.10) | (0.11) | (0.11) | (0.09) | (0.08) | (0.09) | (0.13) | (0.10) |
| has a partner, no children | 0.25 | 0.53*** | 0.74*** | -0.04 | 0.24 | 0.46*** | -0.33* | 0.20 | 0.36* |
|  | (0.17) | (0.16) | (0.20) | (0.16) | (0.14) | (0.13) | (0.14) | (0.20) | (0.16) |
| no partner, has children | 0.24* | 0.17 | 0.22 | 0.13 | 0.25* | 0.38*** | -0.04 | -0.04 | -0.01 |
|  | (0.11) | (0.10) | (0.11) | (0.11) | (0.10) | (0.09) | (0.09) | (0.13) | (0.11) |
| South-East (Ref.: North-West) | -0.47 | -0.01 | -2.11*** | 0.45 | -1.13*** | -0.33 | 0.47* | -0.90*** | -0.43 |
|  | (0.26) | (0.21) | (0.26) | (0.23) | (0.20) | (0.18) | (0.18) | (0.25) | (0.22) |
| *Interactions between "South-East" and:* | | | | | | | | | |
| has a partner and children | -0.22 | -0.27* | -0.20 | -0.23 | -0.26* | -0.51*** | 0.08 | -0.29 | -0.30* |
|  | (0.15) | (0.13) | (0.14) | (0.15) | (0.13) | (0.12) | (0.12) | (0.17) | (0.15) |
| has a partner, no children | 0.19 | -0.18 | -0.35 | 0.20 | -0.20 | -0.34 | 0.45* | -0.07 | -0.06 |
|  | (0.24) | (0.22) | (0.25) | (0.23) | (0.21) | (0.18) | (0.19) | (0.27) | (0.24) |
| no partner, has children | 0.04 | -0.05 | -0.10 | -0.26 | -0.31* | -0.27* | 0.06 | -0.10 | -0.08 |
|  | (0.15) | (0.14) | (0.15) | (0.16) | (0.14) | (0.12) | (0.13) | (0.18) | (0.15) |
| *Age (Ref.: 50-54)* | | | | | | | | | |
| age 55-59 | 0.23* | 0.45*** | 0.47*** | -0.03 | 0.00 | -0.19* | -0.45*** | -0.09 | -0.26** |
|  | (0.11) | (0.09) | (0.10) | (0.10) | (0.08) | (0.08) | (0.08) | (0.10) | (0.09) |
| age 60-64 | 0.32** | 0.44*** | 0.51*** | 0.08 | 0.05 | -0.16* | -0.40*** | -0.02 | -0.17 |
|  | (0.10) | (0.09) | (0.09) | (0.10) | (0.08) | (0.07) | (0.07) | (0.10) | (0.09) |
| age 65-69 | 0.38*** | 0.33*** | 0.36*** | 0.11 | 0.14 | -0.09 | -0.34*** | 0.01 | -0.02 |
|  | (0.10) | (0.08) | (0.09) | (0.10) | (0.08) | (0.08) | (0.07) | (0.10) | (0.09) |
| age 70-74 | 0.35*** | 0.27** | 0.25** | 0.19 | 0.24** | 0.11 | -0.14 | 0.24* | 0.23* |
|  | (0.10) | (0.08) | (0.09) | (0.10) | (0.08) | (0.08) | (0.08) | (0.10) | (0.09) |
| age 75-79 | 0.21* | 0.01 | 0.07 | 0.14 | 0.20* | 0.15 | 0.03 | 0.43*** | 0.29** |
|  | (0.10) | (0.09) | (0.09) | (0.10) | (0.09) | (0.08) | (0.08) | (0.11) | (0.10) |
| age 80-85 | 0.08 | -0.17 | -0.08 | 0.17 | 0.16 | 0.38*** | 0.28*** | 0.43*** | 0.38*** |
|  | (0.10) | (0.09) | (0.09) | (0.11) | (0.09) | (0.08) | (0.08) | (0.12) | (0.10) |
| Female | 0.28*** | 0.21*** | 0.27*** | 0.54*** | 0.40*** | 0.64*** | 0.32*** | 0.43*** | 0.32*** |
|  | (0.04) | (0.03) | (0.03) | (0.04) | (0.03) | (0.03) | (0.03) | (0.04) | (0.03) |
| *Education (Ref.: low)* | | | | | | | | | |
| medium | 0.13 | 0.25*** | 0.19*** | 0.19** | 0.10 | -0.00 | -0.06 | 0.10 | -0.01 |
|  | (0.07) | (0.05) | (0.06) | (0.07) | (0.06) | (0.05) | (0.05) | (0.08) | (0.07) |
| high | 0.17** | 0.43*** | 0.23*** | 0.24*** | 0.18*** | -0.07 | -0.25*** | 0.09 | -0.13* |
|  | (0.06) | (0.05) | (0.05) | (0.05) | (0.05) | (0.05) | (0.04) | (0.07) | (0.06) |
| *Working status (Ref.: Retired)* | | | | | | | | | |
| working | 0.21** | 0.38*** | 0.25*** | -0.03 | -0.10* | -0.29*** | -0.15*** | -0.73*** | -0.15** |
|  | (0.07) | (0.06) | (0.06) | (0.06) | (0.05) | (0.04) | (0.04) | (0.06) | (0.05) |
| other | -0.08 | -0.09 | -0.06 | -0.03 | -0.02 | -0.02 | 0.18*** | -0.07 | -0.02 |
|  | (0.06) | (0.05) | (0.06) | (0.06) | (0.05) | (0.05) | (0.05) | (0.07) | (0.06) |
| Household income | 0.02 | -0.00 | 0.01 | -0.00 | 0.00 | 0.01* | -0.01* | 0.02 | 0.02 |
|  | (0.01) | (0.00) | (0.01) | (0.01) | (0.01) | (0.01) | (0.01) | (0.01) | (0.01) |
| Self-rated health | -0.07** | -0.10*** | -0.03 | -0.02 | -0.02 | 0.14*** | 0.30*** | 0.05* | 0.15*** |
|  | (0.02) | (0.02) | (0.02) | (0.02) | (0.02) | (0.02) | (0.02) | (0.02) | (0.02) |
| Diagnosed illness | -0.04 | 0.18*** | 0.10* | 0.22*** | 0.11** | 0.06 | 0.03 | 0.04 | 0.01 |
|  | (0.05) | (0.04) | (0.04) | (0.04) | (0.04) | (0.03) | (0.03) | (0.05) | (0.04) |
| Gali | -0.08 | -0.07 | -0.09* | -0.02 | 0.01 | 0.28*** | 0.22*** | 0.24*** | 0.13** |
|  | (0.05) | (0.04) | (0.04) | (0.04) | (0.04) | (0.03) | (0.03) | (0.05) | (0.04) |
| Respondent or close | 0.13 | 0.07 | 0.22** | -0.01 | 0.02 | 0.12* | -0.13* | 0.03 | 0.04 |
| relatives tested positive | (0.08) | (0.07) | (0.08) | (0.07) | (0.06) | (0.06) | (0.06) | (0.08) | (0.07) |
| *Country (Ref.: Austria)* | | | | | | | | | |
| Germany | -0.65** | -0.66*** | -0.91*** | 0.33 | -0.44** | -0.87*** | -0.70*** | -0.20 | -0.65*** |
|  | (0.20) | (0.16) | (0.22) | (0.17) | (0.15) | (0.14) | (0.14) | (0.19) | (0.16) |
| Sweden | -0.25 | -0.08 | -1.35*** | -5.58*** | -0.78*** | -0.21 | -1.54*** | -0.83*** | -0.27 |
|  | (0.21) | (0.16) | (0.22) | (0.34) | (0.15) | (0.14) | (0.15) | (0.18) | (0.17) |
| Netherlands | 0.03 | -0.39* | -1.23*** | -6.05*** | -0.70*** | -0.62*** | -1.15*** | 0.04 | -0.19 |
|  | (0.25) | (0.18) | (0.24) | (0.60) | (0.17) | (0.15) | (0.17) | (0.23) | (0.19) |
| Spain | 1.24*** | 1.25*** | 1.28*** | 1.02*** | 1.84*** | 1.50*** | 1.42*** | 2.03*** | 1.65*** |
|  | (0.19) | (0.15) | (0.13) | (0.16) | (0.15) | (0.13) | (0.13) | (0.22) | (0.19) |
| Italy | 0.73*** | 0.72*** | 1.06*** | 1.56*** | 1.92*** | 1.58*** | 2.23*** | 2.15*** | 1.55*** |
|  | (0.14) | (0.12) | (0.11) | (0.15) | (0.13) | (0.11) | (0.12) | (0.16) | (0.14) |
| France | -0.73*** | 0.24 | -1.10*** | -0.22 | -0.76*** | -0.20 | 0.66*** | -0.15 | -0.20 |
|  | (0.21) | (0.17) | (0.22) | (0.17) | (0.16) | (0.14) | (0.14) | (0.19) | (0.17) |
| Denmark | -0.21 | 1.63*** | -0.57* | -5.52*** | -0.11 | -0.96*** | -1.50*** | -0.66*** | -0.50** |
|  | (0.22) | (0.21) | (0.23) | (0.30) | (0.16) | (0.14) | (0.15) | (0.19) | (0.17) |
| Greece | 0.49*** | 0.33** | 1.39*** | -1.47*** | -0.19* | 0.72*** | 0.91*** | 1.17*** | 0.50*** |
|  | (0.13) | (0.11) | (0.10) | (0.10) | (0.09) | (0.09) | (0.09) | (0.11) | (0.10) |
| Switzerland | -0.16 | 0.48** | -1.02*** | -2.85*** | -0.51** | 0.30* | -0.39** | 0.56** | 0.20 |
|  | (0.22) | (0.17) | (0.23) | (0.18) | (0.16) | (0.15) | (0.14) | (0.22) | (0.18) |
| Belgium | -0.20 | -0.01 | -1.29*** | -1.30*** | -0.21 | 0.03 | 0.01 | 0.77*** | 0.46** |
|  | (0.21) | (0.17) | (0.22) | (0.17) | (0.16) | (0.14) | (0.14) | (0.22) | (0.18) |
| Israel | 0.30 | -0.54*** | 0.72*** | 0.40* | 0.40** | 0.69*** | 0.43*** | 0.68*** | 0.84*** |
|  | (0.17) | (0.13) | (0.13) | (0.15) | (0.13) | (0.12) | (0.12) | (0.16) | (0.16) |
| Czech Republic | -0.26* | 0.22* | 0.81*** | 1.57*** | 0.75*** | 0.14 | -0.10 | 0.63*** | -0.03 |
|  | (0.12) | (0.11) | (0.10) | (0.14) | (0.10) | (0.09) | (0.09) | (0.11) | (0.10) |
| Poland | 0.57*** | 0.30** | 1.44*** | 0.93*** | 0.68*** | 0.53*** | 0.05 | 0.78*** | 0.59*** |
|  | (0.14) | (0.11) | (0.11) | (0.12) | (0.10) | (0.09) | (0.09) | (0.11) | (0.11) |
| Luxembourg | -0.77*** | 0.31 | -0.96*** | 1.77*** | 0.41* | 0.71*** | 0.14 | 1.46*** | 0.62** |
|  | (0.22) | (0.19) | (0.24) | (0.24) | (0.20) | (0.17) | (0.15) | (0.32) | (0.21) |
| Hungary | 0.19 | 0.05 | 2.50*** | -0.36* | 0.57*** | 1.09*** | 0.99*** | 0.44** | 1.24*** |
|  | (0.17) | (0.15) | (0.25) | (0.15) | (0.15) | (0.14) | (0.13) | (0.16) | (0.19) |
| Slovenia | 0.18 | 1.18*** | 0.38*** | 0.54*** | 1.11*** | 1.91*** | -0.75*** | 1.86*** | 1.52*** |
|  | (0.13) | (0.12) | (0.09) | (0.11) | (0.10) | (0.11) | (0.09) | (0.15) | (0.12) |
| Estonia | 0.38** | 0.58*** | 0.28** | -2.52*** | 0.78*** | 0.80*** | -0.65*** | 0.30** | 0.83*** |
|  | (0.12) | (0.11) | (0.09) | (0.11) | (0.10) | (0.09) | (0.09) | (0.10) | (0.11) |
| Croatia | 0.39** | 0.51*** | 1.48*** | -1.64*** | 0.38*** | 0.96*** | 0.70*** | 1.43*** | 1.10*** |
|  | (0.15) | (0.13) | (0.13) | (0.12) | (0.11) | (0.11) | (0.10) | (0.16) | (0.14) |
| Lithuania | 0.74*** | 0.64*** | 1.06*** | 1.02*** | 1.12*** | 1.34*** | 0.20* | 1.12*** | 1.15*** |
|  | (0.16) | (0.13) | (0.12) | (0.15) | (0.12) | (0.11) | (0.10) | (0.14) | (0.14) |
| Bulgaria | 0.51** | -0.41** | 1.01*** | 0.19 | 1.17*** | 0.03 | 0.60*** | -0.00 | -0.15 |
|  | (0.17) | (0.13) | (0.14) | (0.15) | (0.14) | (0.11) | (0.12) | (0.13) | (0.12) |
| Cyprus | 0.68** | 1.77*** | -0.26 | -0.86*** | 0.64*** | 1.61*** | 1.55*** | 1.50*** | 1.21*** |
|  | (0.22) | (0.26) | (0.14) | (0.17) | (0.17) | (0.18) | (0.17) | (0.25) | (0.22) |
| Finland | -0.03 | 0.70*** | -0.80*** | -5.11*** | -1.36*** | -0.32* | -1.19*** | -0.47* | -0.39* |
|  | (0.22) | (0.18) | (0.23) | (0.30) | (0.16) | (0.14) | (0.15) | (0.19) | (0.17) |
| Latvia | 0.31 | -0.07 | 0.85*** | -3.77*** | -0.29* | 0.55*** | -0.45*** | 0.66*** | 0.83*** |
|  | (0.16) | (0.13) | (0.13) | (0.18) | (0.12) | (0.12) | (0.11) | (0.15) | (0.15) |
| Malta | 1.23*** | 1.07*** | 1.38*** | -0.46** | 1.84*** | 1.72*** | 1.30*** | 1.79*** | 1.63*** |
|  | (0.21) | (0.17) | (0.15) | (0.15) | (0.19) | (0.14) | (0.13) | (0.21) | (0.20) |
| Romania | 0.63*** | 0.07 | 1.26*** | 0.69*** | 1.42*** | 1.34*** | 2.40*** | 1.42*** | 1.29*** |
|  | (0.15) | (0.12) | (0.12) | (0.14) | (0.13) | (0.11) | (0.15) | (0.15) | (0.14) |
| Constant | 1.46*** | 0.41 | 2.01*** | 0.04 | 1.10*** | -0.51** | -0.98*** | 1.67*** | 0.83*** |
|  | (0.26) | (0.21) | (0.26) | (0.24) | (0.21) | (0.19) | (0.19) | (0.26) | (0.23) |
| N | 33097 | 33101 | 32926 | 27986 | 27968 | 32829 | 32606 | 32488 | 32504 |

Note: Estimated coefficients (effects on log-odds) from separate logistic regression models for each outcome. Standard errors in parentheses. *** p<0.001; *** p<0.01; *** p<0.05. Estimates correspond to the AMEs represented in Fig. 7 in the main manuscript. A set of dummy variables for the week of interview are also included but coefficients are not shown to save space.

**Table S.8 – Multinomial logistic regression model for COVID-19 vaccine acceptance that includes an interaction between the explanatory variable (kin availability) and country groups, full results**

| Independent variables | Outcome categories | |
| --- | --- | --- |
|  | (Ref.: vaccinated/willing to get the vaccine) | |
|  | not willing | undecided |
|  | to get vaccinated |  |
| *Close kin availability (Ref.: no partner, no children)* |  |  |
| has a partner and children | -0.73*** | -0.25 |
|  | (0.17) | (0.26) |
| has a partner, no children | -0.62* | -0.35 |
|  | (0.29) | (0.46) |
| no partner, has children | -0.16 | 0.29 |
|  | (0.17) | (0.27) |
| South-East (Ref.: North-West) | 0.48 | 1.21*** |
|  | (0.26) | (0.34) |
| *Interactions between "South-East" and:* | |  |
| has a partner and children | 0.37 | -0.22 |
|  | (0.21) | (0.29) |
| has a partner, no children | 0.27 | -0.10 |
|  | (0.36) | (0.50) |
| no partner, has children | 0.20 | -0.48 |
|  | (0.21) | (0.30) |
| *Age (Ref.: 50-54)* | |  |
| age 55-59 | -0.18 | 0.07 |
|  | (0.12) | (0.12) |
| age 60-64 | -0.20 | -0.18 |
|  | (0.11) | (0.12) |
| age 65-69 | -0.30** | -0.29* |
|  | (0.12) | (0.12) |
| age 70-74 | -0.48*** | -0.60*** |
|  | (0.12) | (0.13) |
| age 75-79 | -0.56*** | -0.90*** |
|  | (0.13) | (0.14) |
| age 80-85 | -0.46*** | -1.08*** |
|  | (0.13) | (0.15) |
| Female | -0.04 | -0.08 |
|  | (0.05) | (0.05) |
| *Education (Ref.: low)* | |  |
| medium | -0.11 | -0.01 |
|  | (0.09) | (0.10) |
| high | -0.39*** | -0.29** |
|  | (0.08) | (0.09) |
| *Working status (Ref.: Retired)* |  |  |
| working | 0.21** | 0.06 |
|  | (0.08) | (0.08) |
| other | 0.56*** | 0.32*** |
|  | (0.07) | (0.08) |
| Household income | -0.06*** | -0.15*** |
|  | (0.02) | (0.03) |
| Self-rated health | 0.12*** | 0.08* |
|  | (0.03) | (0.03) |
| Diagnosed illness | -0.17** | -0.22*** |
|  | (0.06) | (0.06) |
| Gali | 0.15* | 0.08 |
|  | (0.06) | (0.07) |
| Respondent or close | -0.28*** | -0.10 |
| relatives tested positive | (0.05) | (0.05) |
| *Country (Ref.: Austria)* | |  |
| Germany | -0.45* | -0.89*** |
|  | (0.18) | (0.27) |
| Sweden | -1.71*** | -1.62*** |
|  | (0.38) | (0.48) |
| Netherlands | -1.29*** | -1.69** |
|  | (0.37) | (0.61) |
| Spain | -2.78*** | -4.74*** |
|  | (0.32) | (1.01) |
| Italy | -1.89*** | -1.67*** |
|  | (0.18) | (0.20) |
| France | -0.26 | -0.08 |
|  | (0.19) | (0.24) |
| Denmark | -1.79*** | -2.96*** |
|  | (0.32) | (0.73) |
| Greece | -1.08*** | -0.42** |
|  | (0.15) | (0.15) |
| Switzerland | 0.74*** | 1.14*** |
|  | (0.18) | (0.23) |
| Belgium | -1.16*** | -1.54*** |
|  | (0.23) | (0.36) |
| Israel | -2.11*** | -2.21*** |
|  | (0.33) | (0.47) |
| Czech Republic | -0.78*** | -0.73*** |
|  | (0.15) | (0.17) |
| Poland | -0.38** | -0.21 |
|  | (0.14) | (0.15) |
| Luxembourg | -0.48 | -1.18** |
|  | (0.26) | (0.45) |
| Hungary | -0.54** | -1.64*** |
|  | (0.21) | (0.36) |
| Slovenia | -0.16 | 0.29* |
|  | (0.13) | (0.14) |
| Estonia | -0.53*** | -0.14 |
|  | (0.13) | (0.14) |
| Croatia | -0.35* | 0.25 |
|  | (0.16) | (0.15) |
| Lithuania | 0.12 | 0.08 |
|  | (0.14) | (0.16) |
| Bulgaria | 1.97*** | 2.30*** |
|  | (0.16) | (0.17) |
| Cyprus | -1.22*** | -0.41 |
|  | (0.30) | (0.27) |
| Finland | -1.42*** | -0.93** |
|  | (0.28) | (0.32) |
| Latvia | 0.74*** | 1.00*** |
|  | (0.15) | (0.16) |
| Malta | -2.66*** | -3.83*** |
|  | (0.36) | (0.72) |
| Romania | 1.50*** | 1.33*** |
|  | (0.13) | (0.15) |
| Constant | -1.50*** | -2.33*** |
|  | (0.27) | (0.36) |
| N | 27432 | |

Estimated coefficients (effects on log-odds) from a multinomial logistic regression model for the three-level categorical outcome vaccine acceptance (reference = vaccinated/willing to get the vaccine). Standard errors in parentheses. *** p<0.001; *** p<0.01; *** p<0.05. Estimates correspond to the AMEs represented in Fig. 8 in the main manuscript. A set of dummy variables for the week of interview are also included but coefficients are not shown to save space. Data are from SHARE Corona Survey 2 (June-August 2021).
